# VPS4 is a dynamic component of the centrosome that regulates centrosome localization of γ-tubulin, centriolar satellite stability and ciliogenesis

**DOI:** 10.1038/s41598-018-21491-x

**Published:** 2018-02-20

**Authors:** Carolyn Ott, Dikla Nachmias, Shai Adar, Michal Jarnik, Shachar Sherman, Ramon Y. Birnbaum, Jennifer Lippincott-Schwartz, Natalie Elia

**Affiliations:** 1 Janelia Research Campus, Howard Hughes Medical Institute, Ashburn, Virginia USA; 2 Department of Life Sciences, and National Institute for Biotechnology in the Negev (NIBN), Ben Gurion University of the Negev, Beer Sheva, Israel; 3 Eunice Kennedy Shriver National Institute for Child Health and Human Development, Bethesda, Maryland USA; 4 Department of Life Sciences, Ben Gurion University of the Negev, Beer Sheva, Israel

## Abstract

The hexameric AAA ATPase VPS4 facilitates ESCRT III filament disassembly on diverse intracellular membranes. ESCRT III components and VPS4 have been localized to the ciliary transition zone and spindle poles and reported to affect centrosome duplication and spindle pole stability. How the canonical ESCRT pathway could mediate these events is unclear. We studied the association of VPS4 with centrosomes and found that GFP-VPS4 was a dynamic component of both mother and daughter centrioles. A mutant, VPS4^EQ^, which can’t hydrolyze ATP, was less dynamic and accumulated at centrosomes. Centrosome localization of the VPS4^EQ^ mutant, caused reduced γ-tubulin levels at centrosomes and consequently decreased microtubule growth and altered centrosome positioning. In addition, preventing VPS4 ATP hydrolysis nearly eliminated centriolar satellites and paused ciliogensis after formation of the ciliary vesicle. Zebrafish embryos injected with GFP-VPS4^EQ^ mRNA were less viable, exhibited developmental defects and had fewer cilia in Kupffer’s vesicle. Surprisingly, ESCRT III proteins seldom localized to centrosomes and their depletion did not lead to these phenotypes. Our data support an ESCRT III-independent function for VPS4 at the centrosome and reveal that this evolutionary conserved AAA ATPase influences diverse centrosome functions and, as a result, global cellular architecture and development.

## Introduction

The AAA ATPase VPS4 is part of the endosomal sorting complex required for transport (ESCRT) machinery, which is acutely recruited to selected cellular locations to execute membrane constriction and fission. Although the ESCRT machinery in eukaryotic cells is composed of 5 different protein families (ESCRT 0, I, II, III and VPS4) the minimal functional, evolutionarily conserved unit of the ESCRT machine is composed of the ESCRT III protein CHMP4B (Snf 7 in yeast) and VPS4^1^. According to the current model, ESCRT-III proteins remodel membranes by assembling into filaments that constrict membranes away from the cytoplasm^1–4^. The ATPase activity of VPS4, is essential for active membrane remodeling^1,5–9^. The ESCRT III / VPS4 machine has been shown to facilitate numerous membrane remodeling events in cells including multivesicular body (MVB) formation, release of retroviruses from the cell surface, and scission of daughter cells during the last stages of cytokinesis^10–12^. More recently, ESCRT III and VPS4 were also shown to be involved in resolving problems during nuclear pore formation, stitching together the nuclear envelope during mitotic exit and sealing small ruptures in the plasma membrane^12,13^.

ESCRT III and VPS4 have also been implicated in centrosome function. Depletion of VPS4 or ESCRT III components alters centrosome numbers and size and causes defects in polar spindle formation and chromosome segregation during cell division^14^. In addition, endogenous VPS4 concentrates at centrosomes and spindle pole bodies in HeLa cells^14^. In *Chlamydomonas reinhardtii*, VPS4 and five other ESCRT proteins were isolated from flagellar transition zones- structures that emanate from a specialized form of the centrosome^15^. Transition zone localization of VPS4 was further demonstrated by immuno-staining. Little is known about the targeting and dynamics of ESCRT III and VPS4 to centrosomes. Moreover, it is not clear whether VPS4 only functions at specialized centrosomes, such as spindle poles and basal bodies, or if VPS4 is an integral centrosome component that governs many essential centrosome functions.

The centrosome coordinates diverse processes in cells including intracellular trafficking, cell shape and polarity, cell cycle, and differentiation. It is composed of a mother and a daughter centriole that are surrounded by pericentriolar material (PCM). γ-tubulin and many other PCM proteins are layered around the centrioles, creating a platform for continuous microtubule (MT) nucleation and anchoring, which serves as the main microtubule-organizing center (MTOC) in cells^16^. Unlike sites of conventional ESCRT machinery function, the centrosome is a non-membranous cellular organelle. Because all current models of ESCRT machinery function involve membranes^1^, it is unclear how VPS4 and the ESCRT machinery could function at the centrosome.

In this study, we investigated the dynamics and function of VPS4 in centrosomes. Using structured illumination microscopy (SIM), Airyscan microscopy and live cell confocal imaging we determined that VPS4 dynamically associates with both mother and daughter centrioles. In contrast to other sites of VPS4 activity, we found no evidence that ESCRT III proteins partner with VPS4 at centrosomes. A dominant negative VPS4 mutant incapable of ATP hydrolysis, carrying a Glu to Gln mutation at position 228 (referred to here as VPS4^EQ^), accumulated at the centrosome and exhibited slower turnover than wild-type (WT) VPS4. We used this mutant to investigate the consequences of VPS4 activity and dynamics on centrosome composition and function. We found that while some centrosome components are not affected by VPS4^EQ^ expression, γ-tubulin levels are dramatically reduced. This phenotype was completely abolished upon hijacking VPS4^EQ^ from the centrosome, indicating that centrosomal γ-tubulin levels are specifically regulated by the activity of VPS4 at the centrosome. Reduced centrosomal γ-tubulin levels correlated with a decrease in the γ-tubulin ring complex (γTuRC) interacting protein, NEDD1, and resulted in fewer microtubules emanating from centrosomes. Further, centrosome positioning was altered, centriolar satellites were substantially reduced, and ciliogenesis was impaired. Taken together these results indicate a fundamental role for VPS4 in centrosomes and suggest that VPS4 activity at centrosomes is different from its established ESCRT III-dependent membrane remodeling activities.

## Results

### Spatiotemporal characterization of VPS4 at the centrosome of interphase cells

**Figure 1:**
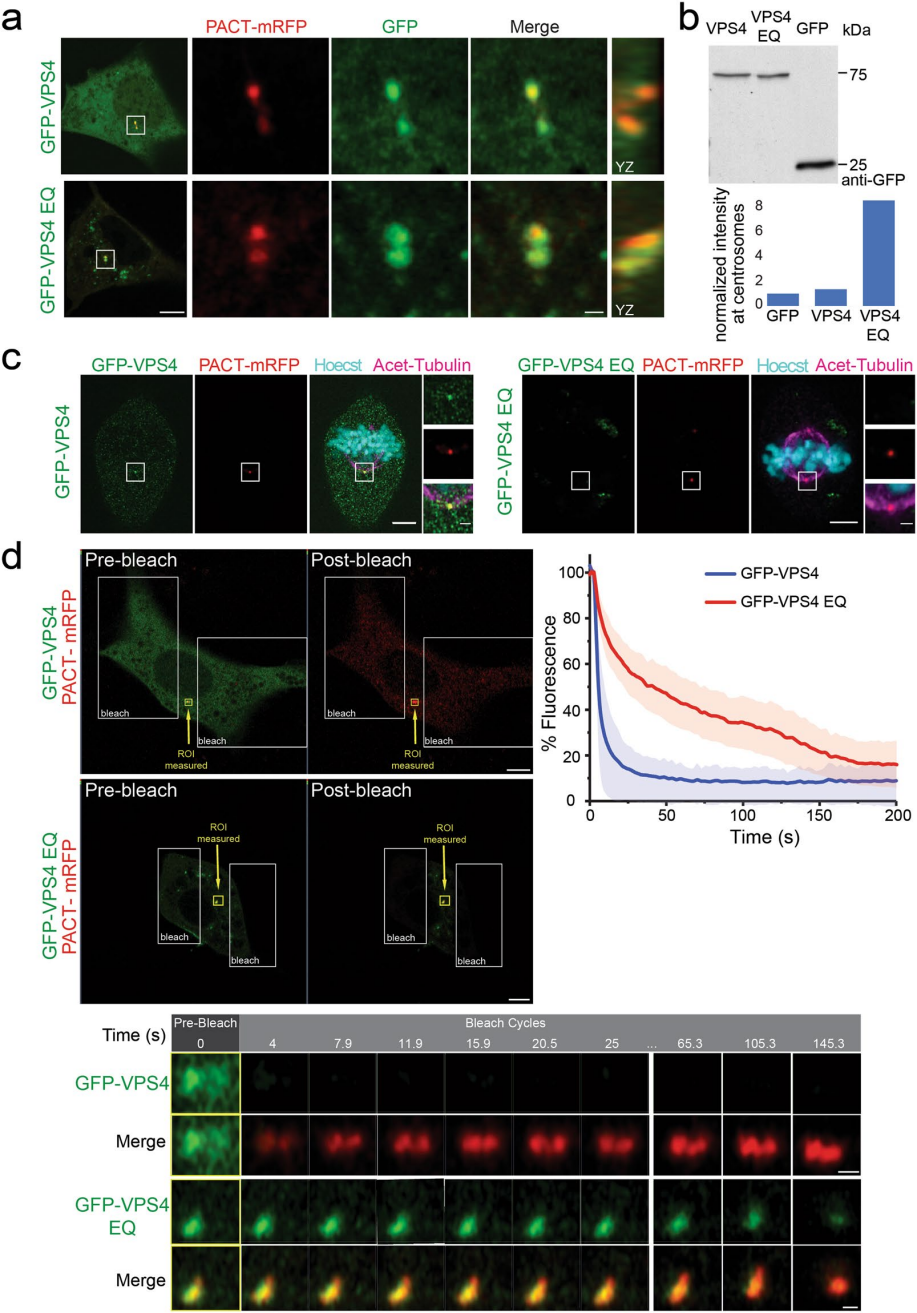
Localization and dynamics of VPS4 at the centrosomes of interphase cells. (**a**) NIH3T3 cells were transfected with the centrosome marker, PACT-mRFP, and GFP-VPS4 or GFP-VPS4^EQ^ and imaged by Airyscan confocal microscopy. Images were processed to reveal the sub-diffraction localization of the proteins. Both the wild-type and the ATPase deficient VPS4 localize to the centrosome. Left to right: entire cell (scale, 5 μm), zoomed-in images (white box) of: PACT (red), VPS4 (green), an overlay and a YZ projection (scale, 0.5 μm). (**b**) Top panel: NIH3T3 cells transfected with GFP, GFP-VPS4 or GFP-VPS4^EQ^ were harvested and lysed 24 h post transfection and subjected to western blot analysis using anti-GFP antibodies. Equal total protein amounts were loaded (see also supplementary Fig. S9). GFP-VPS4 and GFP-VPS4^EQ^ express at similar levels. Bottom panel: Cells expressing GFP, GFP-VPS4, or GFP-VPS4^EQ^ together with PACT-TagBFP were fixed and stained with anti-centrin. Z stacks of centrosomes were collected based on centrosomal markers using Airyscan microscopy. GFP intensity at the centrosome was quantified as described in materials and methods. Shown are average intensity values that were normalized to average GFP intensity obtained in control cells. GFP n = 56, GFP-VPS4 n = 35, GFP-VPS4^EQ^ n = 52 from 2 independent experiments. (**c**) Cells transfected with PACT-mRFP and GFP-VPS4 or GFP-VPS4^EQ^ were grown in high glucose media, fixed, and stained with Hoecst and antibodies to acetylated tubulin. GFP-VPS4^EQ^ expressing cells in anaphase or metaphase like the one shown here were rare. Scale, whole cells, 5 μm; zoomed-in image, 1 μm. (**d**) iFRAP recordings of NIH3T3 cells expressing PACT-mRFP and either GFP-VPS4 or GFP-VPS4^EQ^ were performed using Airyscan Confocal imaging in Fast mode. A single plane was imaged and then two regions on either side of the centrosome (covering most of the cell) were photobleached (white frame box). Image capture alternated with photobleaching for 90–100 time points. The intensity of the GFP signal at the centrosome was quantified using the location of the PACT-mRFP marker to generate the analysis region (small yellow frame box). Intensity was normalized to the pre-bleach intensity and the mean+/− the standard deviation was plotted for GFP-VPS4 and GFP-VPS4^EQ^ (top right panel). Bottom panel: zoomed-in images taken from yellow frame boxes in the corresponding upper panels are shown. GFP-VPS4 n = 11, GFP-VPS4^EQ^ n = 5. Scale, upper panel, 5 μm; bottom panel, 0.5 μm.

VPS4 has previously been shown to localize to centrosomes and spindle poles^14^, however, its dynamics at the centrosome in live cells have not been investigated. To study the localization and dynamics of VPS4 at the centrosome, NIH3T3 cells were co-transfected with GFP-VPS4 (which has previously been used to study VPS4 dynamics in other cellular contexts^17^) and the centrosomal marker PACT-mRFP (the centrosomal targeting motif of pericentrin) and imaged at subdiffraction resolution using a confocal Airyscan microscope. Notably, cells were subjected to mild starvation (low glucose) in order to enrich the population of cells in interphase and to reduce indirect phenotypes resulting from VPS4 function during cell division. In order to avoid overexpression artifacts, only low expressing cells in which the cellular distribution of GFP-VPS4 was mostly cytosolic were selected (supplementary Fig. S1a). In the majority of the cells (68%), GFP-VPS4 was detected in PACT-mRFP labeled centrosomes (Fig. 1a), consistent with the reported localization of endogenous VPS4^14^. Notably, GFP-VPS4 was found at both mother and daughter centrioles in live cells.

We next asked whether the ATPase activity of VPS4 is required for its centrosome localization by transfecting NIH3T3 cells with the ATP-locked mutant, GFP-VPS4^EQ^. Over expressed VPS4^EQ^ is known to localize to, and induce the accumulation of, MVBs^18,19^. Here too, only low expressing cells in which a cytosolic pool of VPS4^EQ^ was clearly observed were selected (supplementary Fig. S1a). In non-dividing cells, the enrichment of GFP-VPS4^EQ^ at PACT-mRFP centrosomes was six-fold greater than the wild type protein, under similar expression levels (Fig. 1b). This indicates that GFP-VPS4^EQ^ accumulates at centrosomes and suggests that the ATPase activity of VPS4 affects its association with centrosomes. In dividing cells grown in high glucose, GFP-VPS4 localized to the spindle poles (Fig. 1c), as previously reported^14^. However, we were unable to find dividing cells with GFP-VPS4^EQ^ at the spindle poles (Fig. 1c). In fact, GFP-VPS4^EQ^ expressing cells in anaphase and metaphase were extremely rare, implying that preventing VPS4 ATP hydrolysis disrupted or delayed mitotic entrance.

To assess the dynamics of VPS4 and VPS4^EQ^ in centrosomes we applied a fluorescent recovery after photobleaching (FRAP) assay to cells transfected with PACT-mRFP and either GFP-VPS4 or GFP-VPS4^EQ^. Using confocal spinning disk microscopy GFP-tagged proteins at the centrosome were photobleached and recovery of fluorescence intensity was measured. While GFP-VPS4^EQ^ quickly bleached and slowly recovered, we observed only a small drop in fluorescence intensity after the bleach phase in cells expressing GFP-VPS4 (supplementary Fig. S1b). Under similar expression levels the pool of GFP-VPS4 at the centrosome is six-fold less than GFP-VPS4^EQ^ (Fig. 1b) and yet we observed only slight bleaching of the wild-type protein. A possible explanation is that ATP hydrolysis by GFP-VPS4 promotes extremely rapid turnover at the centrosome such that almost as soon as a molecule is bleached, it is replaced by a fluorescent protein from the cytoplasm. To further investigate this possibility, we reversed the photobleaching pattern and instead of bleaching the centrosome, we repeatedly bleached the cytoplasmic pool of GFP-VPS4 or GFP-VPS4^EQ^ and monitored the loss of fluorescence at the centrosome (an inverse FRAP, or iFRAP). These experiments were performed using Airyscan Fast-mode microscopy to optimize resolution and sensitivity instead of speed. While the fluorescent centrosomal pool of GFP-VPS4 dropped quickly, GFP-VPS4^EQ^ intensity at the centrosome decreased slowly through the bleaching cycles (Fig. 1d). These results indicate that fluorescent GFP-VPS4 molecules at the centrosome are quickly replaced by bleached cytoplasmic wild-type GFP-VPS4. The inability to hydrolyze ATP reduces the dissociation rate of GFP-VPS4^EQ^ resulting in a slower decay in GFP-VPS4 fluorescence at the centrosome. This data, along with the observation that GFP-VPS4^EQ^ is more enriched at the centrosome than the wild type protein, suggested that VPS4 is dynamically recruited to and released from the centrosome in an ATP hydrolysis dependent manner.

### ESCRT III components do not co-localize with VPS4 at centrosomes

**Figure 2:**
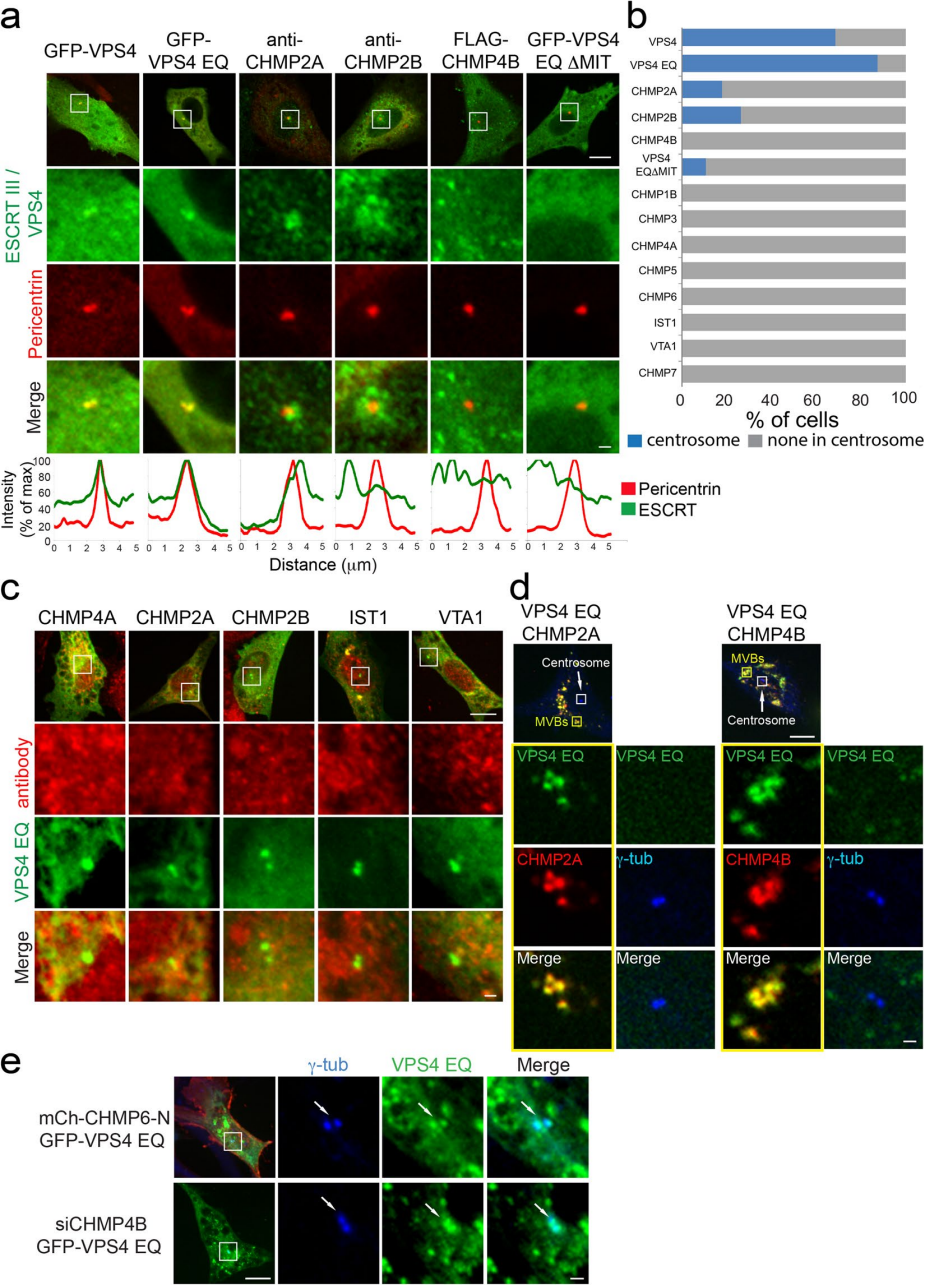
ESCRT-III components do not recruit VPS4 to centrosomes. (**a**) Maximum projection images of representative NIH3T3 cells, transfected with Pericentrin–RFP, and the indicated plasmids or immunostained for the endogenous ESCRT-III proteins CHMP2A or CHMP2B are shown. Top to bottom: entire cell (scale 10 μm), zoomed-in images of the centrosome (white box): ESCRT-III component (green), Pericentrin (red), an overlay (scale, 1 μm) and a line intensity profile of both channels along the centrosome. (**b**) Percentage of cells exhibiting centrosome localization of the indicated proteins (corresponds to Fig. 2a and supplementary Fig. S2a). n > 100, from at least two independent experiments. (**c**) NIH3T3 cells expressing GFP-VPS4^EQ^ were fixed, immunostained with the indicated ESCRT-III antibodies and imaged using a confocal spinning disk microscope. Shown are representative images (data obtained from at least two independent experiments for each protein tested). Top to bottom: entire cell (scale, 10 μm), zoomed-in images (white box) of: ESCRT-III (red), VPS4^EQ^ (green) and an overlay (scale, 1 μm). (**d**) NIH3T3 cells, transfected with GFP-VPS4^EQ^ and the indicated ESCRT III components, were immunostained with anti γ-tubulin and imaged. Shown are images of representative cells. Upper panel: entire cells (scale, 10 μm). Zoomed-in images (marked by squares in upper panel) of MVBs (left; yellow) and the centrosome (right; white) are shown below: VPS4^EQ^ (green), ESCRT III (red), γ-tubulin (blue) (scale 1 μm). Co-transfection with mCherry-CHMP2A n = 12, co- transfection with mCherry-CHMP4B n = 9. (**e**) VPS4 is recruited to the centrosome independent of ESCRT III. NIH3T3 cells were co-transfected with GFP-VPS4^EQ^ and mCherry-CHMP6-N peptide (composed of the first 52 amino acids of CHMP6) (upper panel) or with siCHMP4B and GFP-VPS4^EQ^ (bottom panel). Cells were then fixed, immunostained with γ-tubulin antibodies and imaged. Left to right: entire cell (scale, 10 μm), zoomed-in images (white box) of: γ-tubulin (blue), VPS4^EQ^ (green) and an overlay (scale, 1 μm). White arrows mark the centrosome. CHMP6-N n = 21, siCHMP4B n = 13.

VPS4 is recruited to function at membranes by the ESCRT III complex^5,7,18^ and different ESCRT III components were shown to affect centrosome number and size in dividing HeLa cells^14^. In an effort to identify ESCRT III proteins that partner with VPS4 at the centrosome we first investigated the localization of ESCRT III components to centrosomes. Surprisingly, the majority of ESCRT III components, including the essential ESCRT III component CHMP4B, were not found at the centrosomes of interphase cells (Fig. 2a,b and supplementary Fig. S2a). Similar results were obtained for VTA1, an ESCRT associated protein that binds VPS4. In a small fraction of the cells (less than 30%) the ESCRT III components CHMP2A and CHMP2B were detected in the vicinity of the centrosome (Fig. 2a,b) albeit with a distinct, more peripheral localization pattern compared to GFP-VPS4 (compare line intensity profiles in Fig. 2a).

The absence of centrosome localization observed for ESCRT III could result from the characteristic transient association of these proteins with their site of operation^17,20^. The VPS4^EQ^ mutant inhibits ESCRT III depolymerization, thus trapping the ESCRT III components at the membrane^18^. We therefore assayed the centrosome localization of ESCRT III proteins in cells expressing GFP-VPS4^EQ^. While GFP-VPS4^EQ^ decorated the centrosomes in these cells, no co-localization with endogenous CHMP4A, CHMP2B, IST1 or VTA1 was found at centrosomes (Fig. 2c). CHMP2B, which we occasionally observed associated with the centrosome (Fig. 2a), was not observed in the centrosome of cells expressing GFP-VPS4^EQ^, suggesting that VPS4 can be recruited to centrosomes independent of CHMP2B. To test for centrosome localization of ESCRT III components to which we could not find specific antibodies, we over-expressed tagged versions of different ESCRT III components together with GFP-VPS4^EQ^. As expected, enlarged internal structures (most probably MVBs) accumulated in these cells (Fig. 2d and supplementary Fig. S2b). However, co-expression of almost every ESCRT III subunit together with GFP-VPS4^EQ^ led to trapping of VPS4^EQ^ in these internal structures and to loss of GFP-VPS4^EQ^ from the centrosome (Fig. 2d and supplementary Fig. S2b). Although co-expression of CHMP5-mCherry and GFP-VPS4^EQ^ did not cause loss of VPS4 from centrosomes, it did not lead to accumulation of CHMP5 at centrosomes (supplementary Fig. S2b). Taken together, these data suggest that, unlike VPS4, ESCRT III components may not be an integral part of the centrosome.

### VPS4 centrosome recruitment is independent of ESCRT III

Puzzled by our inability to detect ESCRT III components at the centrosome we wondered if VPS4 might be recruited to centrosomes in the absence of the ESCRT III complex. We have previously shown that expression of a peptide composed of the N terminal region of CHMP6 interferes with ESCRT II-mediated ESCRT III recruitment during cytokinesis, leading to abscission failure^21^. Co-expression of CHMP6 N terminal peptide and GFP-VPS4^EQ^ did not interfere with VPS4 centrosome enrichment (Fig. 2e). GFP-VPS4 also accumulated at centrosomes in cells siRNA depleted of CHMP4B (Fig. 2e). Together this data suggests that the canonical ESCRT pathway components are dispensable for VPS4 recruitment to centrosomes. Interestingly, an ESCRT III-independent role for VPS4 has been suggested in endosomal cholesterol trafficking^22^.

VPS4 contains an N-terminal MIT (Microtubule Interacting and Trafficking) domain that mediates protein-protein interactions, including interactions with components of the ESCRT III complex^1^. Although VPS4 recruitment to centrosomes does not appear to require conventional ESCRT III partners, we wondered if the MIT domain was required for recruitment to centrosomes. To test this, we generated a GFP-VPS4^EQ^ mutant lacking the MIT domain (GFP-VPS4^EQΔMIT^). In contrast to expression of GFP-VPS4 and GFP-VPS4^EQ^, GFP-VPS4^EQΔMIT^ rarely localized to the centrosome (GFP-VPS4, 68%; GFP-VPS4^EQ^, 87%, GFP-VPS4^EQΔMIT^, 11%) (Fig. 2a,b). These results strongly suggest that the MIT domain participates in recruitment of GFP-VPS4^EQ^ to centrosomes. Yet, we found no evidence for any interaction with partners in the canonical ESCRT pathway.

### Centrosome localization of VPS4^EQ^ specifically reduces γ-tubulin at centrosomes

**Figure 3:**
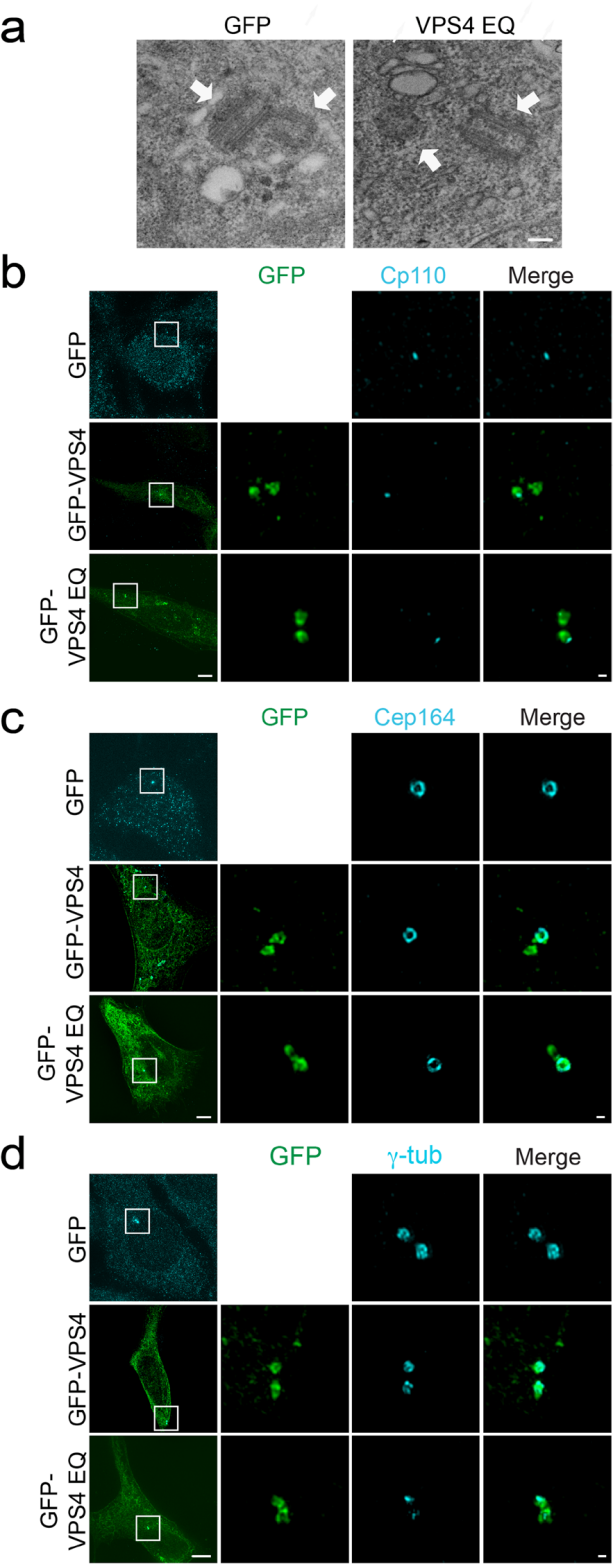
Expression of VPS4^EQ^ causes reduced γ-tubulin staining at centrosomes but does not affect overall centriolar structure. (**a**) NIH3T3 cells grown on gridded coverslips were fixed and imaged to locate cells expressing GFP or GFP-VPS4^EQ^. Cells were then processed for electron microscopy as described in material and methods. Cells selected by fluorescence microscopy were located in the TEM and serial sections were collected to locate the centrosome (supplementary Fig. S3). Scale, 0.2 μm. (**b–d**) The organization of known centrosomal proteins was tested in fixed NIH3T3 cells expressing GFP (control), GFP-VPS4 or GFP- VPS4^EQ^, immunostained with the indicated antibodies. Cells were imaged using 3D SIM. Shown are maximum intensity projections of reconstructed images from representative cells. Each panel shows (from left to right) the entire cell (scale, 5 μm); zoomed-in images (white box) of each channel and a zoomed-in overlay image (scale, 0.2 μm). (**b**) Endogenous CP110 (antibody staining, blue) GFP n = 59, VPS4 n = 10, VPS4^EQ^ n = 20. (**c**) Endogenous Cep164 (antibody staining, blue) GFP n = 10, GFP-VPS4 n = 8, GFP- VPS4^EQ^ n = 15. (**d**) Endogenous γ-tubulin (antibody staining, blue). GFP n = 20, GFP-VPS4 n = 15, GFP-VPS4^EQ^ n = 21. Note that while CP110 and Cep164 are not affected by VPS4^EQ^ expression, γ tubulin staining is severely reduced.

Our data indicate that VPS4 is an active and dynamic component of the centrosome. We therefore set out to investigate how VPS4 activity affects centrosome function. We began by asking whether the reduced dynamics of VPS4^EQ^ at the centrosome influence centrosome organization and morphology in non-dividing cells. To assay the structure of the centriole, we performed correlative light-EM imaging of cells expressing PACT-mRFP and either GFP (control) or GFP-VPS4^EQ^. Expressing cells were first identified using fluorescence microscopy, and then fixed, serially sectioned and imaged by transmission EM. We found 58 centrioles each in GFP and GFP-VPS4^EQ^ expressing cells (summed from 4 experiments) and did not detect any differences in overall structure (Fig. 3a and supplementary Fig. S3). 17 centrioles from each condition were oriented in a way that allowed us to measure their length. The length of centrioles in GFP expressing cells ranged from 364–484 nm with an average of 416 nm. A similar average length (417 nm) was obtained for centrioles from GFP-VPS4^EQ^ expressing cells (range between 373–477 nm), suggesting that the core structure of centrioles is not affected by VPS4.

Depletion of VPS4 in HeLa cells causes cell cycle defects and increases centrosome number. These effects were less severe in other cell types tested^14^. We quantified centrosome numbers in cells grown in high glucose and found no change in centrosome number upon overexpression of GFP-VPS4 or GFP-VPS4^EQ^ in NIH3T3 cells (supplementary Fig. S4).

**Figure 4:**
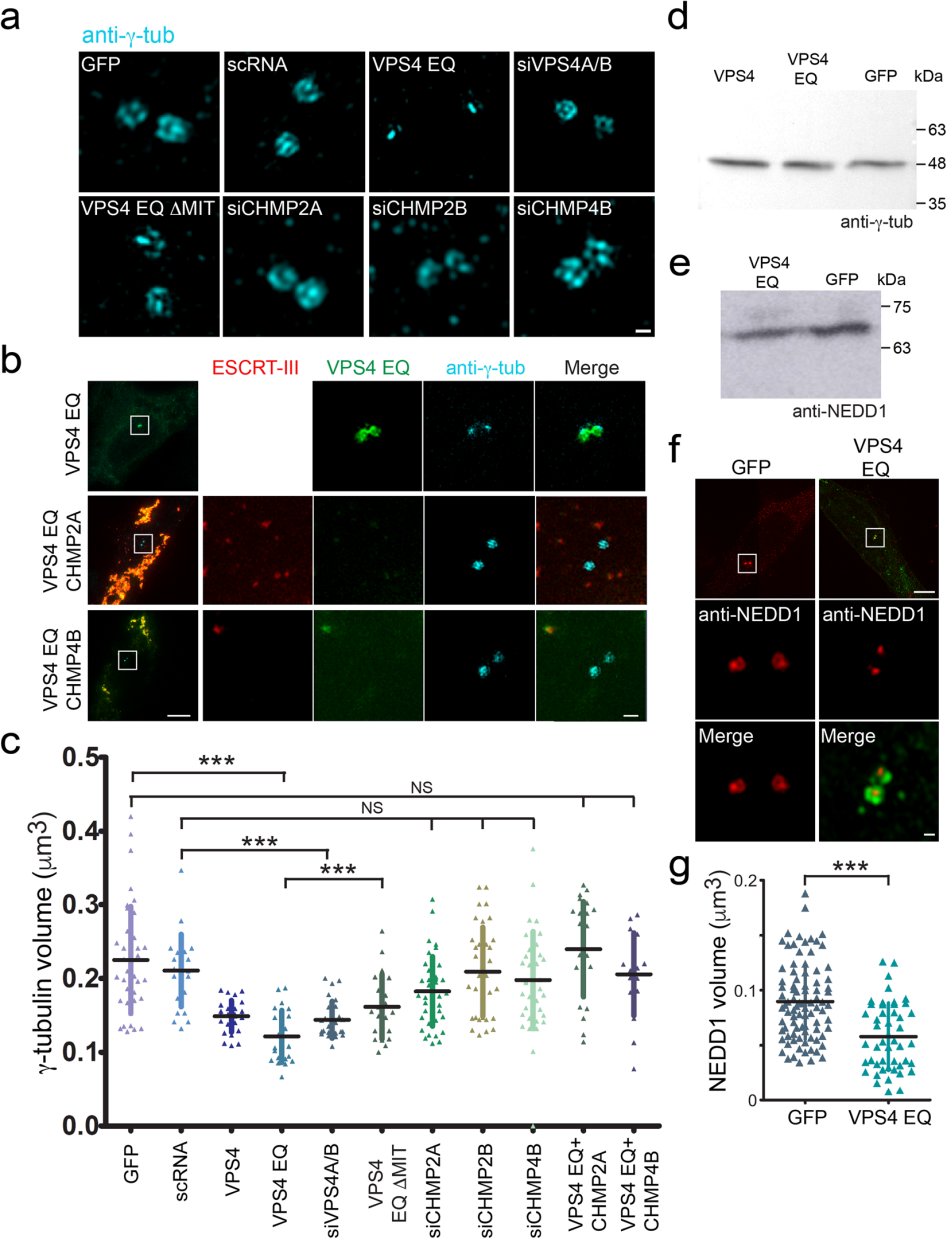
Reduced centrosomal γ-tubulin staining is specifically induced by VPS4 at the centrosome and is unaffected by ESCRT III depletion. (**a**) NIH3T3 cells were transfected with one of the indicated plasmids or siRNA construct. Fixed cells were immunostained for γ-tubulin (blue) and imaged using 3D SIM. Maximum intensity projections of representative cells are shown. GFP n = 20, scRNA n = 17, GFP- VPS4^EQ^ n = 21, siVPS4A/B n = 13, VPS4^EQΔMIT^ n = 11, siCHMP2A n = 20, siCHMP2B n = 12 and siCHMP4B n = 20. Data for each condition was obtained from at least two independent experiments. Scale, 0.2 μm. (**b**) Cells were transfected with GFP-VPS4^EQ^ alone (upper panel) or together with ESCRT-III components (middle and bottom panels). Fixed cells were immunostained for γ-tubulin and imaged using 3D SIM. Maximum intensity projections of reconstructed images from representative cells are shown. Left to right: an overlay image of the entire cell (scale, 10 μm), zoomed in images of the centrosome (white box): ESCRT-III (red), VPS4^EQ^ (green) or γ-tubulin (blue) and an overlay (scale, 0.5 μm). GFP-VPS4^EQ^ n = 21, co-transfection with mCherry-CHMP2A n = 12, co-transfection with mCherry-CHMP4B n = 9. (**c**) 3D volume of centrosomal γ-tubulin structure was calculated in each cell using Volocity image analysis package. Statistical analysis for average volume was calculated using a one-way ANOVA. ***p- value ≤ 0.0001. (**d**) NIH3T3 cells transfected with the indicated plasmids were harvested 24 h post transfection and subjected to western blot analysis using anti-γ-tubulin antibodies. Equal total protein amounts were loaded (see also supplementary Fig. S9). (**e**,**f**) NIH3T3 cells transfected with GFP or GFP-VPS4^EQ^ were either harvested 24 h post transfection and subjected to western blot analysis using anti-NEDD1 antibodies (**e**) (see also supplementary Fig. S9), or fixed and immunostained with anti-NEDD1 antibodies (**f**). Top to bottom in (**f**): an overlay image of the entire cell (scale, 5 μm), zoomed in images of the centrosome (white box): NEDD1 (red) and an overlay (scale, 0.2 μm) GFP n = 46, GFP-VPS4^EQ^ n = 34. (**g**) 3D volume of centrosomal NEDD1 in each cell was calculated using Volocity image analysis package. Statistical analysis for average volume was calculated using t-test. ***p- value ≤ 0.0001.

Next, we used immunofluorescence and SIM to compare the localization of endogenous centrosome components in GFP, GFP-VPS4 and GFP-VPS4^EQ^ expressing cells. While the localization of CP110 and Cep164 at the distal end of the centriole appeared to be unaffected (Fig. 3b,c), staining of the microtubule nucleating protein, γ-tubulin, was altered (Fig. 3d). Expression of GFP-VPS4 moderately reduced γ-tubulin intensity levels at the centrosome, while expression of GFP-VPS4^EQ^ dramatically reduced γ-tubulin intensity and the characteristic ring structure observed by SIM, was completely lost (Fig. 3d). Systematic measurements of the volume of centrosomal γ-tubulin in rendered 3D SIM images showed more than a two-fold decrease in γ-tubulin volume in GFP-VPS4^EQ^ expressing cells, compared to control cells expressing GFP (VPS4^EQ^ 0.109 ± 0.03 µm^3^, GFP 0.23 ± 0.05 µm^3^) (Fig. 4a,c). Depletion of endogenous VPS4 using siRNA also led to a decrease in γ-tubulin volume, but to a smaller degree (siVPS4A/B 0.145 ± 0.02 µm^3^) (Fig. 4a,c). Notably, western blot analysis revealed that total levels of γ-tubulin are not changed upon expression of GFP-VPS4 and GFP-VPS4^EQ^ (Fig. 4d). Therefore, the reduction in centrosome γ-tubulin volume was likely a consequence of altered recruitment or anchoring of γ-tubulin. Centrosome levels of NEDD1, a protein that associates with the γTuRC^23^, were also reduced in cells expressing GFP-VPS4^EQ^ without affecting overall cellular NEDD1 levels (VPS4^EQ^, 0.057 ± 0.026 µm^3^; GFP, 0.0897 ± 0.0347 µm^3^) (Fig. 4e–g). Taken together, our data indicate that VPS4^EQ^ specifically affected components associated with γTURC at the centrosome and did not cause changes in centriole structure.

Our data suggested that conventional ESCRT III partners do not participate in VPS4 function at the centrosome. To test this further we asked whether depletion of ESCRT III leads to the VPS4 induced γ-tubulin phenotype. Consistent with our previous findings, γ-tubulin volume was unaffected in cells depleted of the ESCRT III components CHMP2A or CHMP2B, which were previously shown to mediate VPS4 recruitment to the ESCRT III polymer (siCHMP2A 0.184 ± 0.04 µm^3^, siCHMP2B 0.209 ± 0.06 µm^3^, scRNA 0.21 ± 0.04 µm^3^) (Fig. 4a,c). γ-tubulin volume was also unaffected in cells depleted of the main ESCRT III subunit, CHMP4B^1,5,24^ (siCHMP4B 0.197 ± 0.05 µm^3^) (Fig. 4a,c) and depletion of the MIT domain in VPS4^EQ^ (GFP-VPS4^EQΔMIT^) restored γ-tubulin volume (VPS4^EQΔMIT^ 0.161 ± 0.04 µm^3^). We conclude that although the MIT domain of VPS4 is important for its activity at the centrosome, conventional ESCRT III components are not essential for γ-tubulin recruitment or retention.

To verify that the γ-tubulin phenotype is an outcome of VPS4 function at centrosomes, we took advantage of our previous observation that GFP-VPS4^EQ^ fails to localize to the centrosome upon co-expression with ESCRT III components (Fig. 2d and supplementary Fig. S2b). Under these conditions, GFP-VPS4^EQ^ and ESCRT III were trapped in internal structures and GFP-VPS4^EQ^ was excluded from centrosomes (Fig. 4b). In the presence of this severe MVB phenotype, γ-tubulin volume and morphology were normal (VPS4^EQ^ + CHMP2A, 0.23 ± 0.06 µm^3^; VPS4^EQ^ + CHMP4B, 0.184 ± 0.05 µm^3^) (Fig. 4b,c). Therefore, hijacking GFP-VPS4^EQ^ from the centrosome is sufficient to restore centrosomal γ-tubulin. This strongly suggests that the observed defect in γ-tubulin localization is not an indirect effect of VPS4, but is directly related to VPS4 activity at the centrosome.

### VPS4 activity affects microtubule growth from the MTOC, peri-nuclear centrosome positioning and centriolar satellites

**Figure 5:**
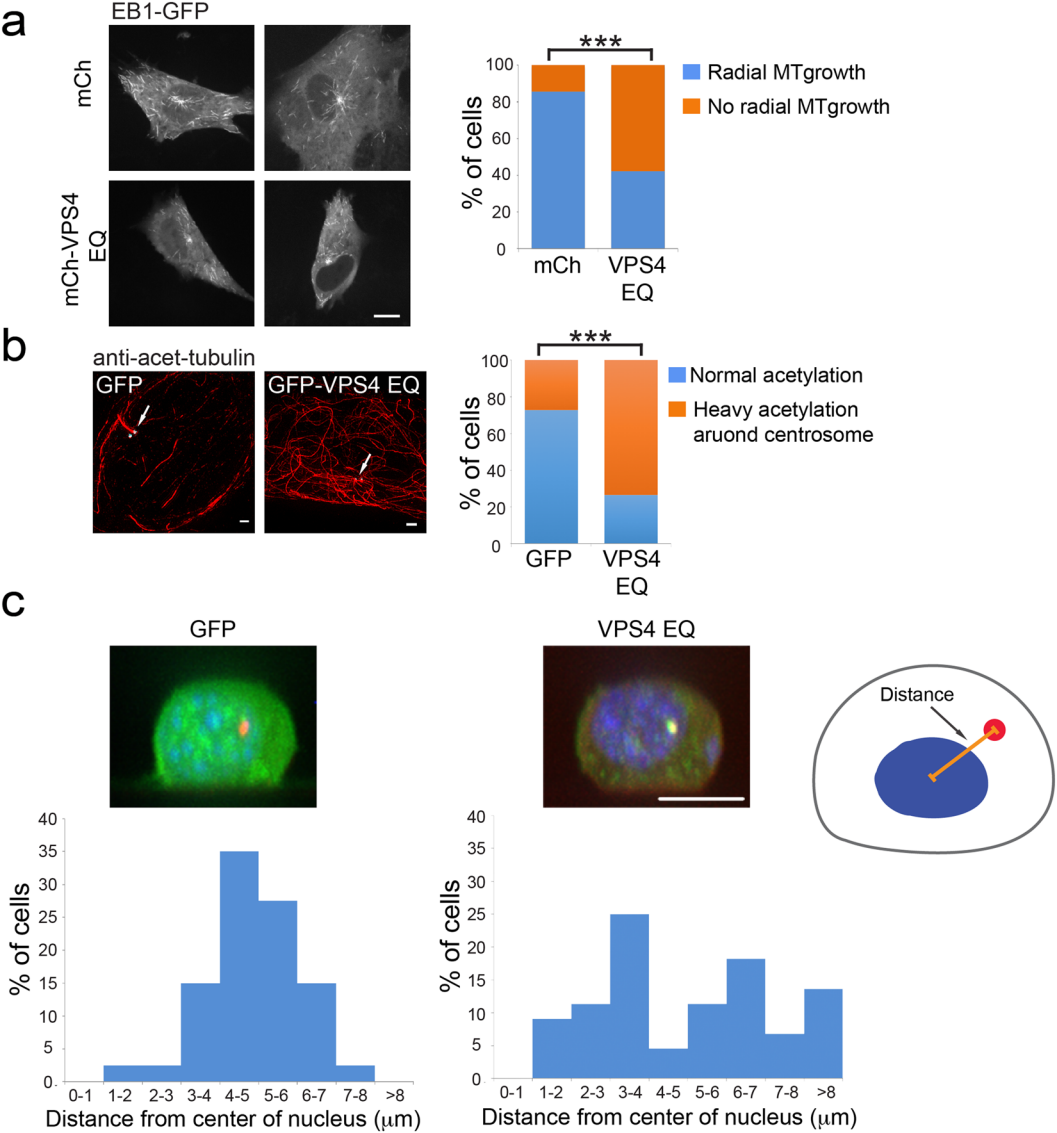
Radial MT growth and centrosome positioning are abnormal upon perturbation of VPS4 ATPase activity. (**a**) NIH3T3 cells were co-transfected with EB1-GFP, a MT plus-end binding protein, and with either mCherry or mCherry-VPS4^EQ^. Shown are time composite of sequential frames acquired for EB1-GFP channel on a spinning-disk confocal microscope at 1 second intervals for 1.5 minutes from representative cells (entire movie series are provided in supplementary Movies 1 and 2). mCherry n = 14, mCherry-VPS4^EQ^ n = 19. Graph on right: percentage of cells in which radial MT growth was observed. Statistical analysis for normal radial MT was calculated using t-test ***p- value ≤ 0.0001. Scale, 10 μm. (**b**) Fixed NIH3T3 cells expressing either GFP or GFP-VPS4^EQ^ were immunostained with anti-acetylated tubulin (red) and anti γ-tubulin (blue) antibodies and imaged in 3D using SIM. Maximum intensity projections of representative images are shown. GFP n = 147, GFP-VPS4^EQ^ n = 115. White arrows indicate the centrosome. Graph on right: percentage of cells exhibiting normal or heavy acetylation. Statistical analysis for normal acetylation was calculated using t-test ***p- value ≤ 0.0001. Scale, 1 μm. (**c**) NIH3T3 cells were transfected with the centrosome marker PACT-mRFP (red) and with either GFP or GFP-VPS4^EQ^ (green). 24 h post transfection cells were plated on fibronectin coated micropatterns (as described in materials and methods), fixed, stained with Hoechst (blue) and imaged in 3D using a spinning disk confocal microscope. Shown are maximum intensity Y projection images of representative cells. The distance between the centrosome and the center of the nucleus (see cartoon on the right) was measured in 3D for each cell as described in materials and methods and plotted in a histogram (bottom panel). GFP n = 45, GFP-VPS4^EQ^ n = 50. Scale, 10 μm.

The centrosome establishes the microtubule organizing center (MTOC) through γ-tubulin mediated nucleation of microtubules that extend throughout the cytoplasm. We therefore tested whether the organization and dynamics of MTs around the MTOC were altered in cells expressing GFP-VPS4^EQ^. Because cells expressing GFP-VPS4 exhibited reduced centrosome γ-tubulin levels (Fig. 3d), we compared the cellular phenotypes of GFP-VPS4^EQ^ transfected cells to cells expressing GFP. Microtubule dynamics were assessed by imaging the MT plus-end binding protein EB1 using spinning disk confocal microscopy. Whereas radial MT growth from the MTOC could be readily detected in control cells, almost no MT growth projecting from the centrosome was detected in the majority of GFP-VPS4^EQ^ cells (Fig. 5a and supplementary Movies 1 and 2). In fact, in some cases the MTOC could not be identified using EB1 labeling alone. Acetylated tubulin staining revealed hyperacetylation of cytoplasmic MTs mainly in the vicinity of the centrosomes (Fig. 5b), consistent with the reduced MT dynamics measured in these cells^25^. Together these results indicate that γ-tubulin function is impaired by the presence of GFP-VPS4^EQ^.

Proper MT growth from the MTOC controls the position of the centrosome in cells^26,27^. To quantitatively determine whether centrosome positioning is altered by GFP-VPS4^EQ^ at the centrosome, we plated NIH3T3 cells expressing PACT-mRFP and either GFP or GFP-VPS4^EQ^ on fibronectin patterned coverslips, and measured the 3-dimensional distance of the centrosome from the center of the nucleus (Fig. 5c). Using this assay, we found the centrosome typically positioned 4–6 microns away from the center of the nucleus in control cells (Fig. 5c, left panel). In contrast, the centrosomes in cells expressing GFP-VPS4^EQ^ appeared more randomly distributed: some centrosomes were located more than 8 microns away while others resided just above or below the nucleus only 2 microns from the center (Fig. 5c, right panel). Therefore, the effect of reduced γ-tubulin microtubule nucleation or anchoring induced by VPS4 ATP hydrolysis extends to centrosome positioning, which governs fundamental cellular behaviors including polarization and cell migration.

**Figure 6:**
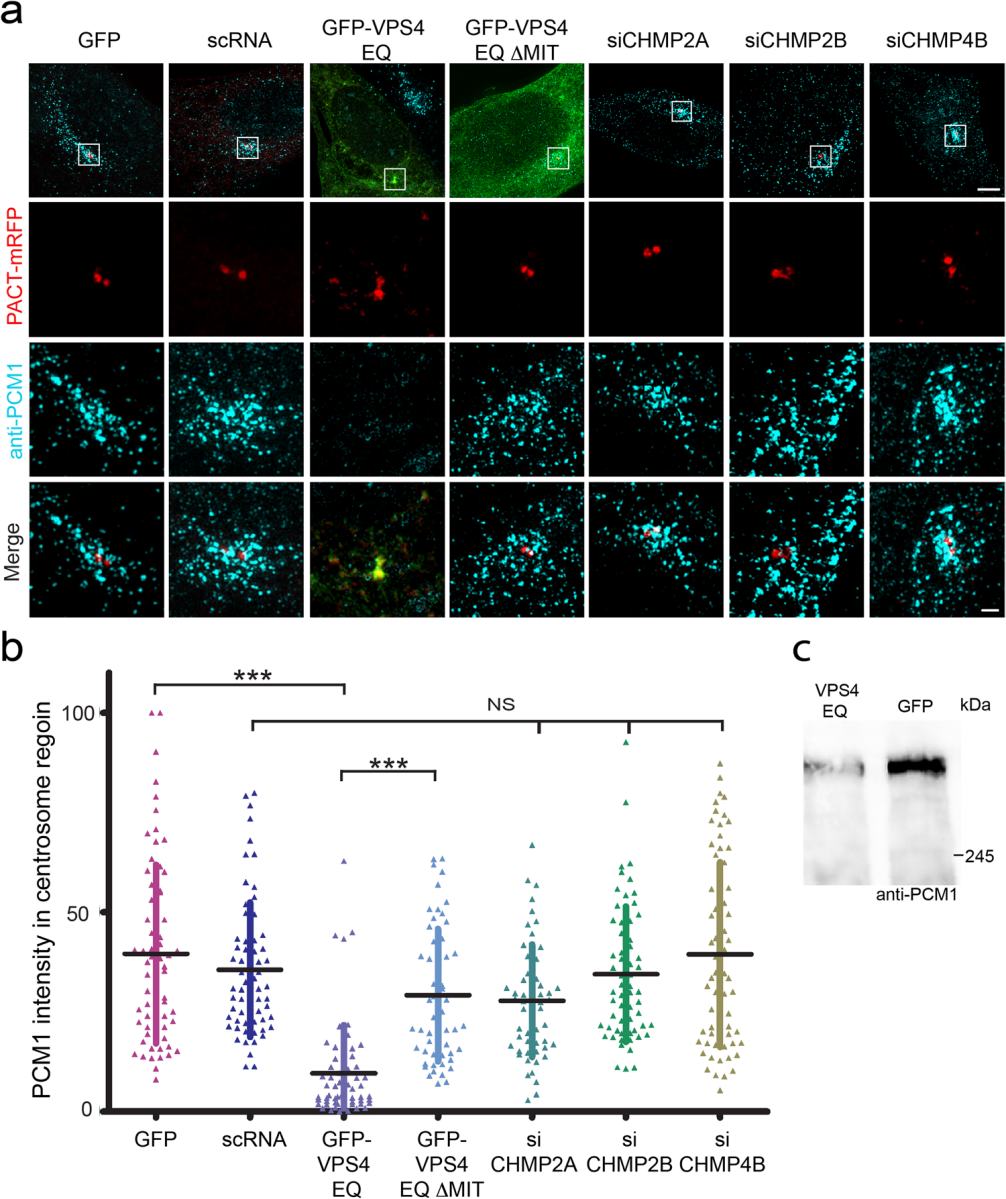
Loss of PCM1 satellites from centrosomes in GFP-VPS4^EQ^ expressing cells. (**a**) NIH3T3 cells were co-transfected with PACT-mRFP (red) together with GFP, GFP-VPS4^EQ^, GFP-VPS4^EQΔMIT^ (green) or siRNA constructs, designed for depletion of CHMP2A, CHMP2B, or CHMP4B. Cells were fixed, immunostained with PCM1 antibodies (blue), and imaged using 3D SIM. Maximum intensity projections of reconstructed images from representative cells are shown. Top to bottom: an overlay image of the entire cell (scale, 5 μm), zoomed-in images of the centrosome (white box): PACT-mRFP (red), PCM1 (blue), an overlay (scale, 0.5 μm). (**b**) Total intensity of PCM1 around the centrosome was measured for the different conditions as described in materials and methods. Statistical analysis was calculated using a one-way ANOVA (***p- value ≤ 0.0001). GFP n = 68, scRNA n = 65, GFP-VPS4^EQ^ n = 61, GFP-VPS4^EQΔMIT^ n = 48, siCHMP2A n = 57, siCHMP2B n = 68, siCHMP4B n = 68. (**c**) NIH3T3 cells transfected with GFP or GFP-VPS4^EQ^ were harvested 24 h post transfection and subjected to western blot analysis using anti-PCM1 antibodies. Equal total protein amounts were loaded (see also supplementary Fig. S9).

The MT network emanating from the MTOC stretches to the cell cortex to form an intracellular transport network. The improper MT growth from the MTOC observed in cells expressing GFP-VPS4^EQ^ could also influence other MT dependent processes. Recently, centriolar satellites have been designated as a fundamental apparatus for assembly and transport of essential protein cargo to centrosomes^28^. Delivery of centriolar satellites to centrosomes was suggested to be MT dependent^29–31^. To test whether centriolar satellites are affected by disruption of VPS4 ATP hydrolysis, we compared the localization of the protein Pericentriolar Material 1 (PCM1), an essential structural component of centriolar satellites, in control GFP and GFP-VPS4^EQ^ expressing cells (Fig. 6a). Remarkably, PCM1 levels in the vicinity of centrosomes were severely reduced in GFP-VPS4^EQ^ expressing cells compared to control cells (VPS4^EQ.^ 9.58 ± 12.13%, GFP 39.56 ± 22.36) (Fig. 6b). Expression of GFP-VPS4^EQΔMIT^ or depletion of CHMP4B, 2B or 2A had little or no effect on PCM1 levels near the centrosome (VPS4 ^EQΔMIT^, 30.48 ± 17.34%; siCHMP4B, 39.45 ± 23.15%; siCHMP2B, 34.48 ± 16.93%; siCHMP2A, 27.83 ± 14.07%; scRNA 35.61 ± 16.82%) (Fig. 6a,b). Notably, the reduced levels of PCM1 observed in GFP-VPS4^EQ^ expressing cells were accompanied by a total reduction in cellular PCM1 levels as observed by western blot analysis (Fig. 6c). Reduction in centriolar satellites was also observed in GFP-VPS4^EQ^ expressing cells using antibodies against the satellite protein Cep290 but was not accompanied by a change in total Cep290 levels (supplementary Fig. S5a–c). Deterioration of the dynamic microtubule network may have prevented proper assembly of centriolar satellites at centrosomes, which in turn lead to degradation of PCM1. Alternatively, VPS4 ATPase activity could have impacted PCM1 levels directly, thus disrupting centriolar satellite assembly.

### VPS4 affects ciliogenesis in mammalian tissue culture cells and in developing zebrafish embryos

**Figure 7:**
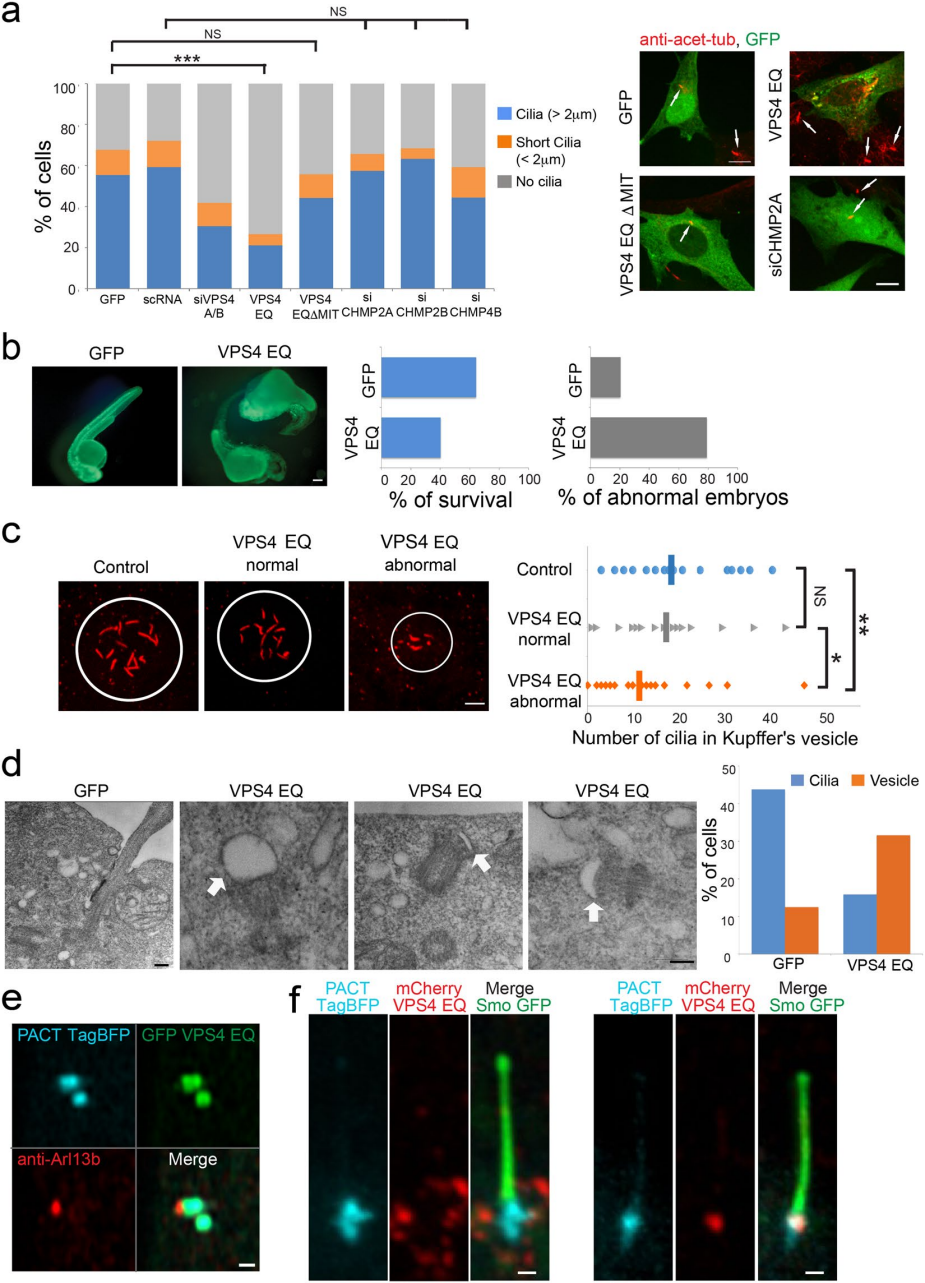
Ciliogenesis is defective in cells with altered VPS4 activity. (**a**) NIH3T3 transfected with the indicated plasmids or siRNA constructs were fixed, immunostained with anti-acetylated antibodies and imaged. The percentage of ciliated cells is displayed in the graph (short cilia ≤ 2 µm, cilia ≥ 2 µm). Statistical analysis was calculated using a one-way ANOVA (***p- value ≤ 0.0001). Maximum intensity projection images of representative cells are shown in the right panel. Arrows indicate cilia (scale, 10 μm). scRNA n = 113, siVPS4A/B n = 103, siCHMP2A n = 64, siCHMP2B n = 173, siCHMP4B n = 68, GFP-VPS4^EQΔMIT^ n = 185. Data was obtained from at least two independent experiments. GFP and GFP-VPS4^EQ^ transfections were repeated in each experiment for reference. For these conditions data was obtained from more than 10 experiments, n ≥ 700. (**b**) Zebrafish embryos were injected with mRNA encoding either GFP or GFP-VPS4^EQ^. 32 h post injection embryos were analyzed for survival (GFP n = 112, GFP-VPS4^EQ^ n = 132). Live embryos were analyzed for developmental defects (GFP n = 71, GFP-VPS4^EQ^ n = 21). Representative images are shown. Scale, 100 μm. (**c**) Embryos (15 h) were fixed and immunostained with acetylated-tubulin antibodies. The number of cilia in the Kupffer’s vesicle of each animal was counted and embryos were categorized as control (not injected or injected with GFP), GFP-VPS4^EQ^ injected developmentally normal, or GFP-VPS4^EQ^ injected with developmental defects. Control n = 41, GFP-VPS4^EQ^ normal n = 17, GFP-VPS4^EQ^ defective n = 31. Statistical analysis was calculated using t-test. *p- value ≤ 0.1, **p- value ≤ 0.05. Scale, 10 μm. (**d**) NIH3T3 cells grown and transfected on gridded coverslips were fixed and imaged using DIC and fluorescence microscopy to locate expressing cells (supplementary Fig. S7). Cells were then treated and serially sectioned as described in material and methods and imaged by TEM. Left panel: representative image of a GFP expressing cells. Second to fourth panels: representative images of cells expressing GFP-VPS4^EQ^. GFP n = 16, GFP-VPS4^EQ^ n = 19. Scale, 0.2 μm. Right panel: percentage of ciliated cells and cells with a docked ciliary vesicle. (**e**) NIH3T3 cells, expressing PACT-TagBFP (blue) and GFP-VPS4^EQ^ (green) and immunostained for the cilia marker Arl13b (red) were imaged using Airyscan confocal microscopy. Scale, 0.5 μm. (**f**) NIH cells transfected with mCherry-VPS4^EQ^ (green) and PACT tagBFP (blue) and Smo GFP (green) were imaged live using Airyscan microscopy. Scale, 0.5 μm.

PCM1 centriolar satellites have previously been shown to be essential for cilia formation^29,31–33^. To test whether VPS4 activity affected ciliogenesis, we compared the percentage of ciliated cells in cells expressing GFP or GFP-VPS4^EQ^ to cells siRNA depleted of VPS4. When grown in the low glucose media used in other experiments, greater than 65% of the GFP transfected cells form primary cilia. We found that both reducing endogenous VPS4 through siRNA depletion and overexpressing the hydrolysis-deficient GFP-VPS4^EQ^ caused a significant decrease in the percentage of ciliated NIH3T3 cells (40% in cells depleted of VPS4, <30% in GFP-VPS4^EQ^ expressing cells) (Fig. 7a and supplementary Fig. S6a). High over-expression levels of WT GFP-VPS4 also led to a decrease in ciliated cells (supplementary Fig. S6a). Consistent with our previous observations, this phenotype was not observed in cells expressing GFP-VPS4^EQΔMIT^ or depleted of the ESCRT III components CHMP4B, 2B or 2A (Fig. 7a).

To substantiate our findings from tissue culture cells we injected mRNA for GFP-VPS4^EQ^ into single cell zebrafish embryos. The survival rate of embryos injected with GFP-VPS4^EQ^ mRNA was less than embryos injected with mRNA for GFP (Fig. 7b). Moreover, many of the GFP-VPS4^EQ^ surviving embryos exhibited severe developmental defects that included head development defects and distorted development of the tail. To test whether cilia formation is impacted by expression of GFP-VPS4^EQ^, we fixed embryos 15 h post fertilization (hpf), stained them for acetylated tubulin and counted the number of cilia in Kupffer’s vesicles^33^. We compared the number of cilia in control embryos to the mutant-expressing phenotypical embryos or to those with developmental defects. There was a small reduction in cilia number on embryos that did not exhibit developmental defects, but this difference was not statistically significant from control. In contrast, embryos exhibiting developmental defects had smaller patches with fewer cilia (Fig. 7c). We therefore concluded that perturbing VPS4 function reduces ciliogenesis in both mammalian tissue culture cells and zebrafish embryos.

Early steps in ciliogenesis have been previously documented by EM^34,35^. To examine which step in ciliogenesis is affected by VPS4, we quantified ciliogenesis in cells expressing GFP or GFP-VPS4^EQ^, using the correlative EM imaging approach described above (Fig. 3a and supplementary Fig. S3). In many control cells a cilium was clearly captured, however, very few cilia were found in GFP-VPS4^EQ^ expressing cells (Fig. 7d and supplementary Fig. S7), confirming our previous observation using fluorescence microscopy (Fig. 7a). Moreover, more than 30% of GFP-VPS4^EQ^ expressing cells had a large vesicle docked at one centriole. This phenotype was three-fold more abundant than in control cells (Fig. 7d). These vesicles appeared to be ciliary vesicles, which are formed early in ciliogenesis after vesicles dock at the distal end of the mother centriole and fuse^34,35^. This observation suggests that cells with defective VPS4 activity are able to undergo early steps in ciliogenesis but fail to advance from this stage to form a mature cilium.

We also observed structures that look like docked ciliary vesicles in fixed cells expressing PACT-TagBFP and GFP-VPS4^EQ^, stained for the cilia marker, Arl13B. Invoking the conventional paradigm, we had anticipated that VPS4 might associate with the pre-ciliary membrane and function to remodel the membrane as the cilium extends; however, we saw no evidence for this. In these cells, GFP-VPS4^EQ^ remained extensively localized in the centrosome and was not transferred to the ciliary membrane (Fig. 7e). These observations support the emerging evidence that VPS4 at the centrosome may function independent of membranes.

In the minor population of GFP-VPS4^EQ^ expressing cells that did form primary cilia, GFP-VPS4^EQ^ was present at the centrosome in many cells, but absent in others (Fig. 7f). On rare occasions, we observed cells with an accumulation of GFP-VPS4^EQ^ at the base of the cilium, likely in the transition zone (supplementary Fig. S6b). This localization may be related to the observed localization of VPS4 at the transition zone in *Chlamydomonas*^15^. It is unclear if VPS4 function at the transition zone is related to the centrosome localization and γ-tubulin induced phenotypes described in this work.

## Discussion

**Figure 8:**
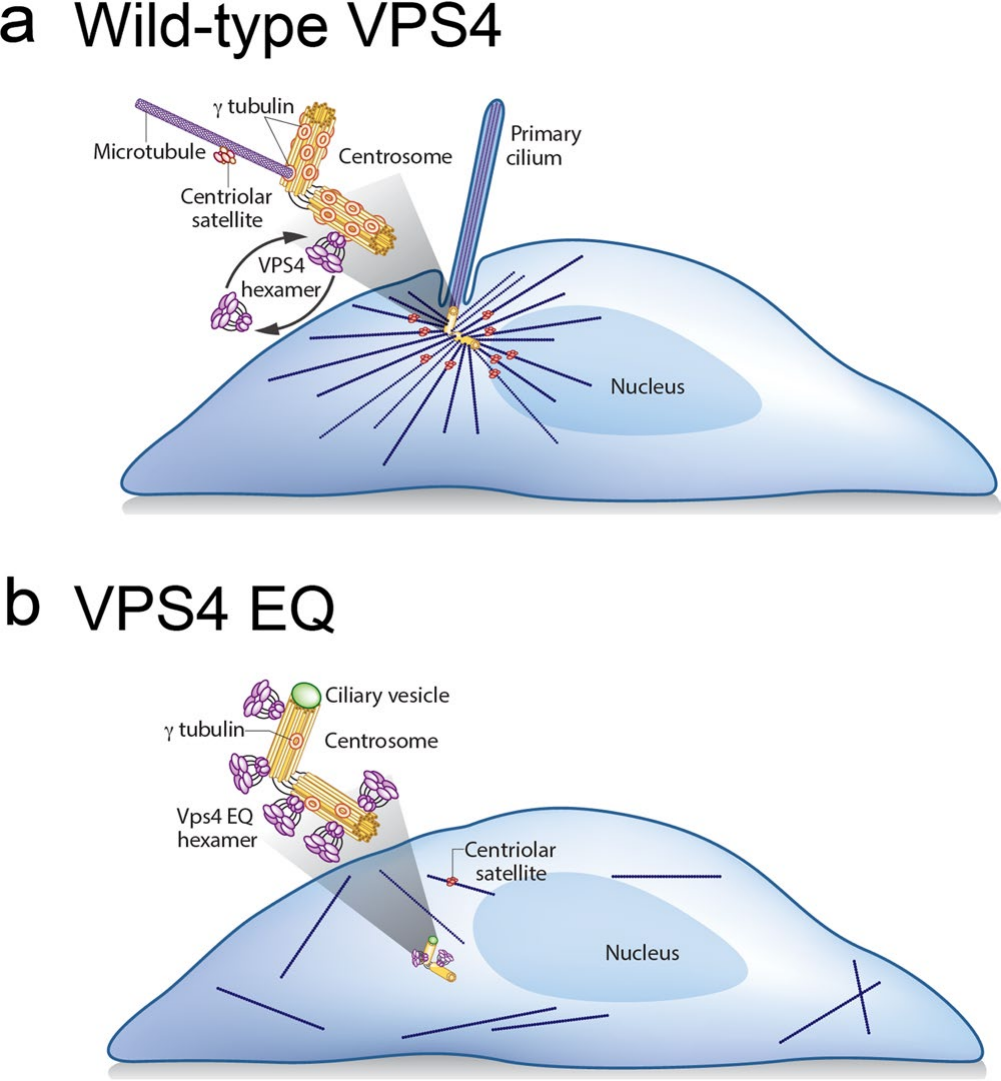
A model for VPS4 function at centrosomes. (**a**) In normal conditions VPS4 dynamically associates with the centrosome. This dynamic VPS4 localization ensures proper γ-tubulin organization and MT growth at the centrosome where it also facilitates ciliogenesis. (**b**) Inhibition of VPS4 dynamic association with the centrosome using the ATP locked mutant VPS4^EQ^, leads to reduced γ-tubulin levels and loss of γ-tubulin ring structure at the centrosome. Consequently, MT growth from centrosomes is impaired, centrosome positioning is misregulated, centriolar satellites are lost, and ciliogenesis is inhibited.

We investigated potential centrosome functions of VPS4 and ESCRT III proteins. Several lines of evidence indicate that VPS4 is recruited to, and functions at centrosomes. In contrast, we found no evidence that ESCRT III proteins act with VPS4 at the centrosome. Interrupting VPS4 dynamics at the centrosome, by inhibiting its ATPase activity in a dominant negative manner, altered centrosome composition and function. The consequences of this included defects in the local processes of ciliogenesis and microtubule nucleation or anchoring, and extended throughout the cytoplasm to impact microtubule stability and cell organization (Fig. 8).

Since the identification of the ESCRT pathway as the machinery that mediates membrane fission to create intraluminal vesicles in MVBs^36^, many additional targets of the ESCRT pathway have been identified^10^. Different proteins trigger the recruitment of early ESCRT proteins at each location, but the ultimate catalytic function is performed by the ESCRT-III VPS4 module that is conserved in Archaea and mammals^1^. The experimental data presented here support the possibility that VPS4 has activities independent of ESCRT III^14,22^. We found no known ESCRT III proteins co-localized with VPS4 at the centrosome under any condition. In a small subset of cells, centrosome localization was observed for the ESCRT III components CHMP2A and CHMP2B. These proteins, however, had a different, more peripheral localization pattern than that observed for VPS4. If ESCRT III proteins recruit VPS4 to the centrosome, we expected that similar to conventional activity sites, depletion of either would have the same phenotype. However, Morita and colleagues reported a reduction in centrosome number in CHMP2A depleted cells and an increase in centrosome number in VPS4 depleted cells^14^. We show that depleting VPS4 resulted in decreased ciliogenesis, while depleting CHMP 2A, 2B, or 4B had little effect. Further, GFP-VPS4^EQ^ localized to the centrosome in cells depleted of CHMP4B, the most essential and evolutionary conserved ESCRT III component. Altogether these data support the model that VPS4 functions independent of ESCRT III at centrosomes.

At conventional recruitment sites, ESCRT III filaments assemble on membranes where they both recruit VPS4 and are the targets of VPS4 catalytic activity^1^. In contrast, we observe VPS4 associated with the membrane-free PCM; even in the presence of the ciliary vesicle. The lack of membranes within centrosomes might preclude ESCRT III filament assembly and therefore necessitate an alternate recruitment partner and target. An additional observation suggests that VPS4 activity at the centrosome may be different from established target locations. On membranes, ESCRT pathway components are recruited, execute membrane remodeling, and disappear. For example, VPS4 is only recruited at the last stage of MVB budding and cytokinesis; there is no stable pool of VPS4 in endocytic vesicles or at the midbody^9,17^. In contrast, we observe that GFP-VPS4 constantly localized to the centrosome and that centrosomal VPS4 is maintained through dynamic, ATP-dependent turnover. Several components of the ESCRT machinery have been previously reported to function outside the canonical ESCRT pathway and to regulate non-membrane cellular processes. For example, CHMP4C participates in cell cycle regulation through binding to the chromosomal passenger complex and ESCRT II is involved in maternal mRNA localization in *Drosophila* oocytes^1,37,38^. We provide evidence that VPS4, can function independent of its evolutionary conserved ESCRT III partners at a non-membrane surface.

The cycle of VPS4 activity at the centrosome parallels VPS4 activity at other cellular locations: VPS4 recruitment is dependent on interactions mediated by the N-terminal MIT domain and ATP hydrolysis precedes the release of VPS4 back into the cytoplasm. The regions in ESCRT III proteins that bind the VPS4 MIT domain, called MIT interacting motifs (MIMs), contain some similar sequences, but these are not highly conserved. It is possible that VPS4 is recruited to centrosomes either by a yet to be identified ESCRT component, or by a non-ESCRT protein carrying a MIM domain. A centrosomal VPS4 target could be highly divergent or contain a domain that is only structurally similar to ESCRT MIMs. In simple BLAST searches of centrosome and cilia proteomes, we were not able to identify any MIM containing proteins but a genome-wide screen for MIM domain proteins or a screen for MIT domain binding proteins could identify candidate VPS4 recruiting partners.

VPS4 is a member of a family of AAA ATPase proteins that includes the microtubule severing proteins spastin, katanin and fidgetin^39^, which have also been shown to localize to centrosomes and spindle poles^40–43^. Disruption of the ESCRT-independent activity of VPS4 at the centrosome echoes phenotypes of disrupting activity of other AAA ATPase family members: a mutation that prevents katanin ATP hydrolysis reduces the volume of γ-tubulin at spindle poles^44^ and changes in the expression levels and activity of fidgetin-like 1 affect ciliogenesis and alter the protein composition of the centrosome^45^. The shared evolutionary origins and similarity of the misregulation consequences at the centrosome raise the possibility that, like other members of the family, VPS4 at the centrosome could target microtubules.

Perturbation of VPS4 function at centrosomes caused several phenotypes, including a significant reduction in centrosome localized γ-tubulin and NEDD1. The ESCRT machinery is known to be involved in protein degradation and in cell division^46–48^. Therefore, changes centrosome size and number caused by ESCRT depletion could be related to problems with cytokinesis or protein degradation. However, this possibility cannot account for the effects of VPS4 reported in this study. *First*, only centrosome localization of γ-tubulin, not the overall cellular levels, change upon expression of GFP-VPS4^EQ^. *Second*, preventing protein degradation through the ESCRT pathway leads to accumulation of proteins at their function site and not to their reduction^49^. *Third*, the effect on γ-tubulin levels depends on VPS4 centrosome localization: sequestering VPS4^EQ^ away from the centrosome by over-expression of ESCRT III proteins completely reverts this phenotype. Therefore, although we do not know the direct target of VPS4, the effect on γ-tubulin recruitment or retention appears to be specific to VPS4 function at the centrosome.

The reduction in NEDD1 and γ-tubulin levels at the centrosome was not correlated with increased centrosome numbers so the observed decrease was not simply a redistribution of material to additional structures. Through interactions with γ-tubulin, NEDD1 recruits the γTuRC to the centrosome and promotes MT anchoring^23,41,50,51^. NEDD1 depletion reduces levels of γ-tubulin at the centrosome and prevents spindle formation^23^. It is therefore possible that the reduced γ-tubulin phenotype we report here (Fig. 4a,c) and our inability to find GFP-VPS4^EQ^ on spindle poles (Fig. 1c) are caused by insufficient levels of NEDD1 at centrosomes in the presence of GFP-VPS4^EQ^. The effect of VPS4 on NEDD1 may also account for some of the mitotic phenotypes reported upon depletion of VPS4^14^. The identification of VPS4 interacting proteins in the centrosome will likely provide insights into this newly identified function of VPS4.

Centriolar satellites are emerging as key modulators of centrosome structure and function, however, their assembly and disassembly are still not fully understood^28^. Dispersion of PCM1 satellites was previously reported upon nocodazole or cold treatment, upon depletion of MT associated proteins and upon dynein dysfunction^29–31^. These findings indicate that an intact MT network is needed for centriolar satellite formation. Like the dispersion phenotypes, the loss in PCM1 satellites observed in our study could be a consequence of the reduced centrosomal γ-tubulin. Yet, the possibility that VPS4 affects centriolar satellites through a MT independent pathway cannot be excluded.

Ciliogenesis is another important process impacted by perturbed VPS4 activity. According to the current model of intracellular ciliogenesis, vesicles dock at the basal body and fuse to one another, giving rise to a structure called the ciliary vesicle (CV) which ensheathes the axoneme as it grows and eventually fuses with the plasma membrane^34,35,52^. Little is known about the transformation of the ciliary vesicle into a growing cilium. Cells expressing VPS4^EQ^ halt ciliogenesis after formation of ciliary vesicles, which results in fewer cilia in both mammalian cells and in developing zebrafish embryos. VPS4 depleted cells also form fewer cilia. Cep164 localization to distal appendages and CP110 uncapping, which are both required for ciliogenesis^35,53^, are not affected by VPS4^EQ^ at the centrosome. Why is VPS4 activity needed for axoneme extension and reshaping of the ciliary vesicle, if VPS4 does not localize to the membrane of the ciliary vesicle? The lack of centriolar satellites is likely a major factor. Several cilia proteins including BBS4 traffic from centriolar satellites to the cilium^54^. PCM1, a scaffold in centriolar satellites^28^, is important for cilia formation^32^ and mislocalization of centriolar satellite proteins interferes with formation of cilia^33^. Like injection of GFP-VPS4^EQ^ mRNA, disrupting PCM1 by injecting antisense morpholinos in zebrafish results in developmental defects and primary cilia phenotypes^33^. It is also possible that problems in microtubule formation and centrosome positioning prevent centrosome anchoring needed for ciliogenesis. Because development requires perception of intracellular signals through cilia localized receptors, VPS4 mediated effects on ciliogenesis may impact complex pattern formation.

Phenotypes were observed upon localization of mutant VPS4, depletion of both VPS4A and B and, to lesser degree, upon overexpression of WT VPS4. This suggests that VPS4 stoichiometry at the centrosome must be tightly regulated in cells. Post-translational modifications, such as phosphorylation, may alter VPS4 affinity to centrosomes and intentionally cause the changes we observe. The VPS4 dependent changes in centrosome composition were accompanied by deregulated centrosome positioning. Because microtubules serve as the highways for vesicle trafficking, VPS4 activity influences both where and how many highways are built, which could have critical consequences. During cell polarization, a transient change in VPS4 could liberate an anchored centrosome and enable it to migrate to a different part of the cell and alter directed intracellular transport. Additionally, in response to acute stress, increased VPS4 at the centrosome could potentially reduce microtubule growth. During development, shifting the amount of VPS4 at the centrosome could delay cilium formation and prevent a cell from responding to extracellular stimuli. In addition to revealing the mechanism of VPS4 function at the centrosome, future studies into the regulation of centrosome-localized VPS4 activity will likely reveal a range of physiological consequences.

## Materials and Methods

### Cell culture and transfection

To enrich the population of interphase cells, NIH3T3 cells were subjected to mild starvation. Cells were either grown in low glucose (1 g/L D-Glucose) DMEM with sodium pyruvate or grown in high glucose (4.5 g/L) DMEM and subjected to low glucose starvation (1 g/L D-Glucose) for at least 24 h. All DMEM media were supplemented with 10% newborn calf serum, 2 mM glutamine. To assess dividing cells and to quantify the effect of VPS4 on centrosome numbers, cells were grown in high glucose DMEM and were not subjected to low glucose starvation. Transfection was carried out using either Lipofectamine 2000 or Lipofectamine 3000 (Life Technologies, Carlbad, CA) according to manufacturer’s guidelines. We found that high overexpression of wild type VPS4 sometimes masked the enrichment of VPS4 at the centrosome. To better visualize wild type VPS4 at the centrosome we reduced the levels of VPS4 plasmid to 1/10 of the total DNA in transfection. This was only done in Fig. 2a,b, Fig. 3b,c. In some cases (Fig. 7e,f, Supplemental Figs 5 and 6b) 1/10 of the total DNA in transfection was used for the VPS4^EQ^ plasmid. In all other cases 250–500 ng plasmid was used for transfection and care was taken to use similar expression levels for all conditions. siRNA transfection cells were transfected twice with 50 nM siRNA duplexes at 24 h interval using Lipofectamine 2000. Cells were harvested and analyzed 48 h after the first transfection. Sequences of siRNA constructs are provided below. Protein depletion via siRNA was verified using western blot analysis (supplementary Fig. S8b).

### Plasmid constructs

VPS4 is VPS4A, unless specified otherwise. VPS4 in pEGFP-C1 vector, VPS4 ATPase-defective mutant (VPS4^EQ^) in pEGFP-C1 vector and a mCherry- CHMP4B in mCherry-C1 vector (Clontech, Mountain View, CA) were previously described^17^. To generate mCherry-VPS4/VPS4^EQ^, sequences were amplified by PCR from GFP-VPS4/VPS4^EQ^ plasmids and cloned into mCherry-C1 vector. To generate GFP-VPS4^EQΔMIT^, VPS4^EQ^ sequence, depleted its first 75 AA was amplified by PCR from GFP-VPS4^EQ^ and cloned into pEGFP-C1 vector. For zebrafish injections eGFP and GFP- VPS4^EQ^ sequences were subcloned into a pCS2 + plasmid kindly provided by Gil Levkowitz (Weizmann Institute of Science, Israel). mCherry-CHMP6-N peptide was previously described in Goliand *et al*.^21^. The following constructs were generous gifts as indicated: Pericentrin–RFP (James Nelson, Stanford University, USA), PACT-mRFP (Sean Munro, MRC, England), Centrin-GFP (Michel Bornens, Institute Curie), CHMP1B-HA (Craig Blackstone, NIH), myc-CHMP7 (Sylvie Urbe, University of Liverpool), Td-Tomato Smoothened (Matt Scott, Stanford University), CHMP4B-Flag and CHMP6-Flag were kind gifts from Wesley Sundquist (University of Utah, USA). PACT-TagBFP, mCherry-CHMP2A CHMP3-GFP, CHMP4A-mCherry, CHMP4C-mCherry, CHMP5-GFP and EB1-GFP were sub-cloned in the Lippincott-Schwartz lab. All constructs were sequenced before use.

### siRNA sequences

siRNA target sequences were designed using IDT (Integrated DNA Technology).

The sequences of siRNA constructs used in this study are:

scRNA: CGUUAAUCGCGUAUAAUACGCGU

siVPS4A: AGCUGAAGGAUUAUUUACGAAACAA

siVPS4B: GGAGCCAAAGAAGCUCUUAAAGAGG siCHMP2A: GAAGAAAUGAUGAAUGACGCAAUUG

siCHMP2B: CUAAGUUGUUUUACACUGAAAUCAG siCHMP4B: GGAGGAGAUGUUAAGCAAGAAGCA

### Immunostaining of tissue culture cells

NIH3T3 cells were fixed 24 h post transfection with either 4% paraformaldehyde (PFA) (15 minutes at room temperature or 10 minutes at 37 °C) or 100% ice-cold methanol (5 minutes, −20 °C). Cells were then permeabilized, blocked and immunostained with the following primary antibodies as specified in text: mouse monoclonal anti α-tubulin antibodies (1:1000, DM1A clone Sigma-Aldrich Cat # T9026), mouse monoclonal anti acetylated tubulin antibodies (1:500 or 1:1000, Thermo Scientific Cat # 32–2700 or 1:1000, Sigma-Aldrich Cat # T7451), mouse monoclonal anti γ-tubulin (1:1000, Sigma-Aldrich Cat #T6557), mouse monoclonal anti NEDD1 (1:100, Abcam Cat # ab57336), rabbit polyclonal anti γ-tubulin antibodies (1:200, Abcam Cat # ab 84355), rabbit polyclonal anti CHMP2A (1:50, Proteintech Group Cat # 10477-1-AP) rabbit polyclonal anti CHMP2B (1:50, Proteintech Group Cat # 12527-1-AP), rabbit polyclonal anti IST1 (1:50, Proteintech Group Cat # 51002-1-AP), rabbit polyclonal anti CP110 (1:50, Proteintech Group Cat # 12780-1-AP) antibodies, rabbit polyclonal anti CHMP4A (1:50, Santa Cruz Biotechnology Cat # SC-67229), rabbit polyclonal anti PCM1 antibodies (1:100, Santa Cruz Biotechnology, Cat # SC-67204), rabbit polyclonal anti Cep164 (1: 200, Abcam Cat # ab106372 or 1:200 Proteintech Group Cat # 22227-1-AP), rabbit polyclonal anti Cep290 (1:100, Abcam Cat # ab84870), mouse anti Centrin (1:500, Millipore clone 20H5, Cat # 04-1624) and rabbit polyclonal anti VTA1 antibodies (1:50, Pierce Biotechnology Cat # PA521831). Secondary antibody staining was performed using Alexa Fluor 405, Alexa Fluor 488, Alexa Fluor 594, or Alexa Fluor 643 (Life Technologies) or Cy3 and Cy5 (Jackson Immuno Research) anti-mouse or anti-rabbit secondary antibodies. Finally, cells were mounted with Fluoromont-G (SouthernBiotech Cat # 00-4958-02) or Vectashield (Vector Labs Cat # H-1000). The specificity of all ESCRT antibodies used in this study was verified by their localization to the intercellular bridge of dividing cells (supplementary Fig. S8a).

### Western blot analysis

Transfected NIH3T3 cells were lysed 24 h post transfection using RIPA lysis buffer [150 mM NaCl, 1% NP-40, 0.5% Deoxycholate, 0.1% SDS, 50 mM Tris (pH 8.0)] supplemented with complete protease inhibitor (Roche Diagnostics, Mannheim, Germany) for 30 minutes at 4 °C. For each condition, total protein concentrations were measured using a BCA Protein Assay Kit (Pierce Biotechnology) and equal total protein amounts were loaded. Membranes were stained with the following antibodies as specified in text: anti-VPS4 (1:750, Sigma-Aldrich Cat # SAB4200025), anti- CHMP2A or CHMP2B (1:800 each, Proteintech Group Cat # 10477–1-AP, Cat # 12527–1-AP), anti-CHMP4B (1:200, Santa Cruz Biotechnology Cat # sc-82556), anti-GFP (1:1000, Applied Biological Materials Inc. Cat # Y050794), anti-NEDD1 (1:500, Abcam Cat # ab57336), anti-γ-tubulin (1:10000, Sigma-Aldrich Cat #T6557), rabbit anti PCM1(1:500, Santa Cruz Biotechnology Cat # SC-67204) or rabbit anti Cep290 (1:5000, Novus Biologicals Cat # 44100002) for 16 h at 4 °C

### Preparation and handling of zebrafish embryos

The animal work performed in this study was approved by the Ben Gurion University Institutional Animal Care and Use Committee. AB wild type zebrafish were maintained and bred at 28 °C according to standard protocols. mRNA encoding for GFP or GFP-VPS4^EQ^ were generated by *in-vitro* transcription from pCS2 + plasmid using mMESSAGE mMACHINE® SP6 Transcription Kit (Thermo Fisher Scientific, Waltham, MA). ~1ng of mRNA was injected into the yolks of one-cell stage wild type embryos using microinjector (World Precision Instrument, Sarasota, FL). Injected and control embryos were kept in 28 °C in Danieau buffer (174 mM NaCl, 21 mM KCl, 12 mM MgSO_4_, 18 mM Ca(NO_3_)_2_, 15 mM HEPES). At 15 hpf embryos were fixed (4% PFA, 8 h at 4 °C) and washed twice in PBST 0.1%. Whole-mount immunostaining was performed according to the protocol described in Stowe 2012^33^. Briefly, embryos were permeabilized with 0.25% trypsin-EDTA (Biological industries) in Hank’s balanced salt solution (Thermo Fisher Scientific, Waltham, MA) for 10 minutes on ice then washed three times for 30 minutes in PBS plus 0.2% Triton X-100. Embryos were blocked in blocking solution (10% fetal bovine serum, 1% BSA and 0.2% Triton X-100 in PBS) for 4 h at room temperature and then incubated with primary and secondary antibodies (anti-acetylated tubulin, 1:1000, and Alexa Flour 594 anti-mouse 1:1000 respectively) for 36 h. Finally, embryos were mounted in 75% glycerol on a microscope slide and imaged by confocal microscopy as described below.

### Confocal microscopy

Fixed NIH3T3 cells, plated in low density were subjected to immunostaining as described above. Z-stacks of cells expressing the designated proteins were imaged using confocal spinning-disk microscope (Marianas; Intelligent Imaging, Denver, CO) with a 63× oil objective (NA, 1.4) and were recorded on an electron-multiplying charge-coupled device (EMCCD) camera (pixel size, 0.079 μm; Evolve; Photometrics, Tucson, AZ). Z-stacks of Kupffer’s vesicle in fixed whole embryos, were collected using a 40× oil objective (NA, 1.3). Image processing and analysis were done using SlideBook version 6 (Intelligent Imaging).

### Live-cell recording and image processing

For live imaging experiments of VPS4 (Fig. 1): Cells were plated on #1.5 Lab Tek II chambers (Nunc, Cat # 155382) or high precision coverslips (BioScience Tools Cat # CSHP-No1.5–10). Images were acquired using Zeiss LSM 880 with Airyscan and Airyscan FAST modes. Live cells were maintained in growth medium and imaged on an incubated stage with 5% CO2 using a Plan-Apochromat 63 × /1.40 Oil DIC objective. Pixel size and z-steps were selected to maximize resolution of the shortest wavelength. Raw images were processed using ZEN software (Carl Zeiss MicroImaging, Jena, Germany). Channel alignment corrections were applied to multi-color images using a channel alignment matrix generated using multi-spectral beads. iFRAP experiments were performed using Airyscan FAST mode. After 4 baseline time points, 2 square regions of interest on either side of the centrosome covering as much of the cell as possible were repeatedly bleached using a 405 laser. 90–100 acquisition and bleaching alternated cycles were performed. Intensity at the centrosome over time was measured in ZEN. Intensities for each condition were averaged across time and the mean ± standard deviation plotted (Fig. 1c). FRAP experiments (supplementary Fig. S1b) were performed on a customized Nikon TiE inverted scope with a Yokogawa spinning-disk scan head (#CSU-X1, Yokogawa) and 2 Photometrics EM-CCD cameras (Evolve 512) using a 100× Plan-Apochromat 1.49 NA oil objective (Nikon) with the additional use of a 1.5× optovar. Fluorescence from two channels was simultaneously collected using the dual camera arrangement.

For EB1 tracking experiments (Fig. 5a and supplementary Movies 1 and 2): NIH3T3 cells plated at low density on a four-well chamber slide (Nunc, Rochester, NY, or ibidi, Martinsried, Germany), were transfected with the plasmids indicated and imaged 24 h later. Images were collected using a fully incubated confocal spinning-disk microscope (Marianas; Intelligent Imaging, Denver, CO) as specified above. Image processing and analysis were done using SlideBook version 6 (Intelligent Imaging). EB1 fluorescence was subjected to a laplacian filter for better visualization and tracking.

### 3D SIM imaging

NIH3T3 cells were plated at 10% density on #1.5 high-precision coverslips (Marienfeld, Lauda-Konigshfen, Germany or Carl Zeiss, Germany Cat # 474030–9000–000) and transfected 24 h later with the indicated plasmids (as described). All samples were subjected to immunostaining as described above. Thin Z-sections (0.11–0.15 μm) of high-resolution images were collected in three rotations for each channel using an ELYRA PS.1 microscope (Carl Zeiss MicroImaging). Images were reconstructed using ZEN. All measurements were performed on reconstructed super-resolution images in ZEN. Line intensity profile measurements were obtained by stretching a curved line along the centrosome.

### Adhesive Micropattern assay

Preparation of micropatterned dishes: fibronectin coated micropatten (700 µm^2^, disc-shape) was generated according to the protocol described in Thery *et al*.^55^. The coated micropatten was stamped on uncoated two well chamber slide (ibidi, Martinsried, Germany) and blocked with an ultrathin layer of poly-L-lysine-grafted-polyethylene glycol (PLL-g-PEG, SurfaceSolutions). NIH3T3 cells co-expressing PACT-mRFP (a centrosome marker) together with either GFP or GFP-VPS4^EQ^ were prepared and plated on the micropatterened wells (24 h post transfection, 50,000 cells per well). 5 h post seeding, cells were fix with 4% PFA fixation and immunostained with Hoechst. 3D Z-stacks were collected using a confocal spinning-disk microscope as described above.

### Data quantitation

To compare the enrichment of proteins at the centrosome (Fig. 1), cells were transfected with the indicated GFP construct, along with PACT-TagBFP and stained after fixation with anti-centrin antibodies. Transfected cells were identified using the 488 channel and z stack ranges were set using the centrosome markers. After automatic Airyscan processing a summed projection was created from all centrosome containing z slices in Image J. In each image, an ROI was selected for each centriole based on the centrosome markers and the summed intensity at each centriole was measured. Average intensity levels in GFP expressing cells were set as 1 and all other average intensity values were normalized accordingly.

γ-tubulin and NEDD1 volumes were measured from Z stack images of cells based on either γ-tubulin or NEDD1 antibody staining using the volume measurements tool in Volocity6 image analysis package (PerkinElmer, Waltham, MA).

Centrosome localization relative to the nucleus was measured by determining the X, Y, Z coordinates of center of the nucleus and the centrosomes using a mask object of each structure applied to a maximum Y projection of the image. The coordinates of the center of the nucleus were subtracted from the centrosome coordinates to calculate the relative distance of the centrosome from the center of the nucleus.

Quantification of centriolar satellites: Cells transfected with the indicated GFP construct and PACT-mRFP, were stained with antibody against PCM1 or Cep290. Centrosomes were located using PACT fluorescence and the total intensity of PCM1 or Cep290 was measured in an area of 0.85 µm^2^ around the centrosome. All measurements were subjected to background subtraction. Intensity values were normalized to intensity of centriolar satellites in the control cells (GFP transfected cells).

### Statistical analysis

Data was analyzed for column statistics with GraphPad Prism version 5.00 for Windows (La Jolla, CA, USA) and Statistica software (Statistica). Statistical significance was determined by t-test or ANOVA as specified in text.

### Correlative Light-Electron Microscopy

1.5 × 10^5^ NIH3T3 cells were plated on fibronectin-coated gridded cover glass (EMS Cat. #0.72265–50) in a 6-well plate and transfected with DNA coding for the centrosome marker, PACT-mRFP, and either GFP or GFP-VPS4^EQ^ using Lipofectamine 2000 as described above in low glucose medium. The following day cells were rinsed in 1 × PBS, and fixed in warm 4% PFA, 1 × PBS at 37 °C for 10 minutes. Coverslips were transferred to imaging chambers and imaged in 1 × PBS using DIC and wide-field microscopy (red and green filters) through a 20× air objective to map the locations of transfected cells on the grid (supplementary Figs S3 and S7). Cells were then removed from the imaging chamber and fixed again in 2.5% Gluteraldehyde, 2% PFA and 2 mM CaCl2 in 0.1 M Cacodylate buffer (pH 7.4) for 15 minutes at room temperature followed by 45 minutes on ice. After rinsing, samples were exposed for 1 minute to 0.15% tannic acid in 0.1 M cacodylate buffer pH 7.4, washed twice in the buffer and post-fixed in 2% OsO_4_ in the same buffer. Samples were extensively washed with water and immunostained o/n with 2% aq. uranyl acetate, dehydrated through series of increasing concentration of ethanol (30%, 50%, 70%, 90%, 3 × 100% anhydrous) and embedded in EMBed 812 epoxy resin (EMS). After resin polymerization, the coverslip was removed by hydrofluoric acid, cells previously imaged by light microscopy identified by their position on the grid, cut out and remounted. Serial ultrathin sections 70–80 nm thick were cut parallel to the plane of the coverslip and mounted on formvar/carbon coated slot (0.5 × 2 mm) EM grids. To enable precise alignment of the block in respect to the knife, thick (approx. 100 nm) film of carbon was evaporated on the surface of the block. Sections were immunostained with 2% uranyl acetate and imaged in FEI Tecnai 20 transmission electron microscope operated at 120 kV. Images were recorded on AMT XR81 wide angle CCD camera.

## Electronic supplementary material

Movie 1

Movie 2

Supplementary figures
