# Supplementary material for: VPS4 is a dynamic component of the centrosome that regulates centrosome localization of γ-tubulin, centriolar satellite stability and ciliogenesis
Source: Sci Rep. 2018 Feb 20;8:3353. doi: 10.1038/s41598-018-21491-x (PMC5820263; doi:10.1038/s41598-018-21491-x)
Supplement: Supplementary file 3 — Supplementary figures [file 41598_2018_21491_MOESM3_ESM.pdf]

VPS4 is a dynamic component of the centrosome that regulates centrosome localization of γ-tubulin, centriolar satellite stability and ciliogenesis

Carolyn Ott[1$,] Dikla Nachmias[2$], Shai Adar[2], Michal Jarnik[3], Shachar Sherman[2], Ramon Birnbaum[4], Jennifer Lippincott-Schwartz[1], Natalie Elia[2*]

a

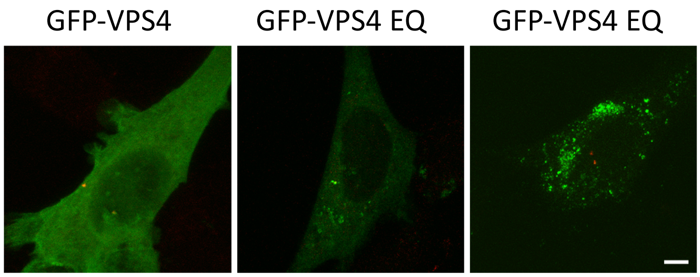

b

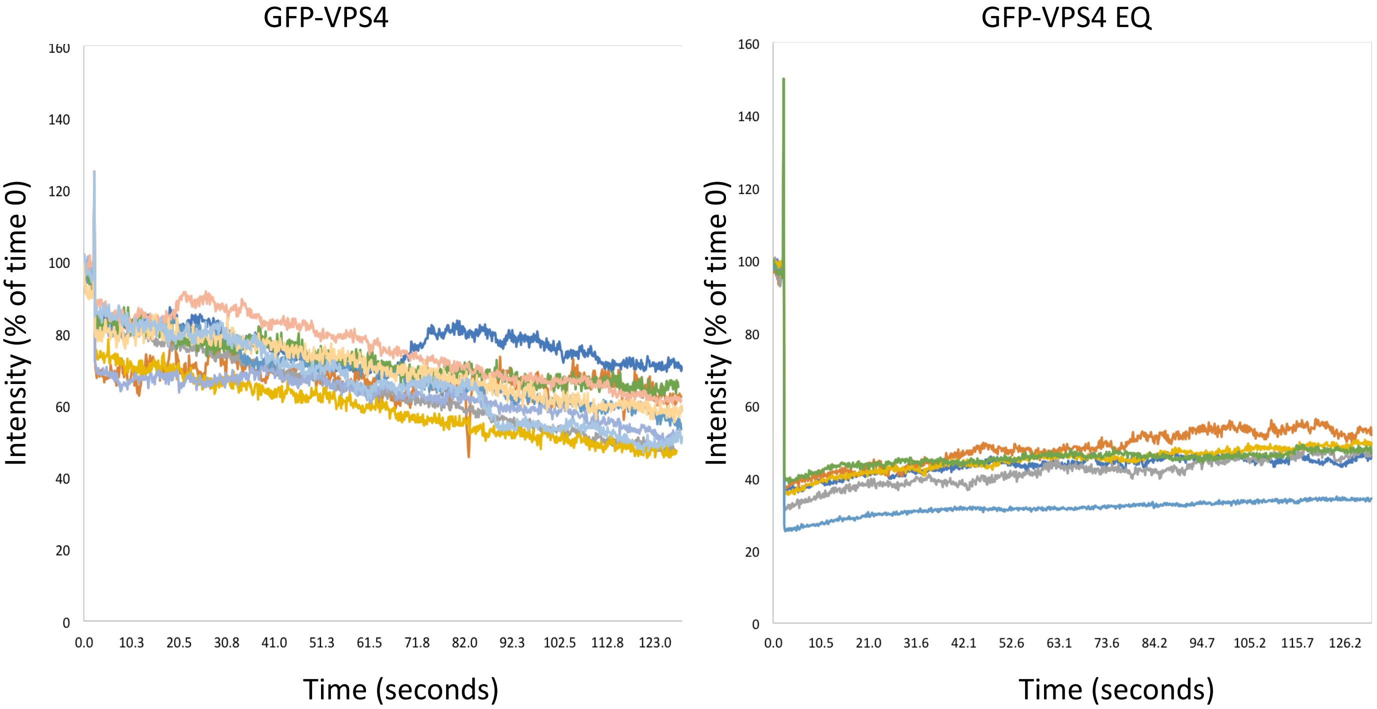

supplementary Fig. 1

Measuring VPS4 dynamics at centrosomes

(a) NIH3T3 cells were transfected with either GFP-VPS4 or GFP-VPS4^{EQ} together with PACT-mRFP. Heterogeneous cellular distribution was observed for VPS4. To avoid over expression artifacts only cells that exhibited mild to low expression levels and that had a significant pool of cytosolic VPS4 were selected for imaging and for further analysis. In cells expressing GFP-VPS4 only cells in which VPS4 was mostly cytosolic were considered suitable for analysis (left panel); in cells expressing GFP-VPS4^{EQ} cells exhibiting both cytosolic and MVB localization of VPS4^{EQ} were considered suitable for analysis (middle panel) while cells exhibiting mostly MVB localization were excluded from analysis (right panel). Scale, 10 µm.

(b) NIH3T3 cells transfected as described in **(a)** were subjected to FRAP analysis. Centrosomes were located based on PACT-mRFP fluorescence and an ROI of 31.5 μm^2 around the centrosome was photobleached once using a 405 laser. Centrosomes were imaged for at least two minutes post photobleaching. Using the signal from PACT-mRFP the location of the centrosome was tracked as the centrosome moved and the fluorescence intensity of GFP was measured overtime. A dual camera setup (see materials and methods) was used to maximize acquisition speed. Total intensity prior to photopleaching (average of 15 time points) was set as 100%. The values over 100% indicate the bleaching event. Traces from individual cells are shown GFP-VPS4 n=10, GFP-VPS4^{EQ} n=6.

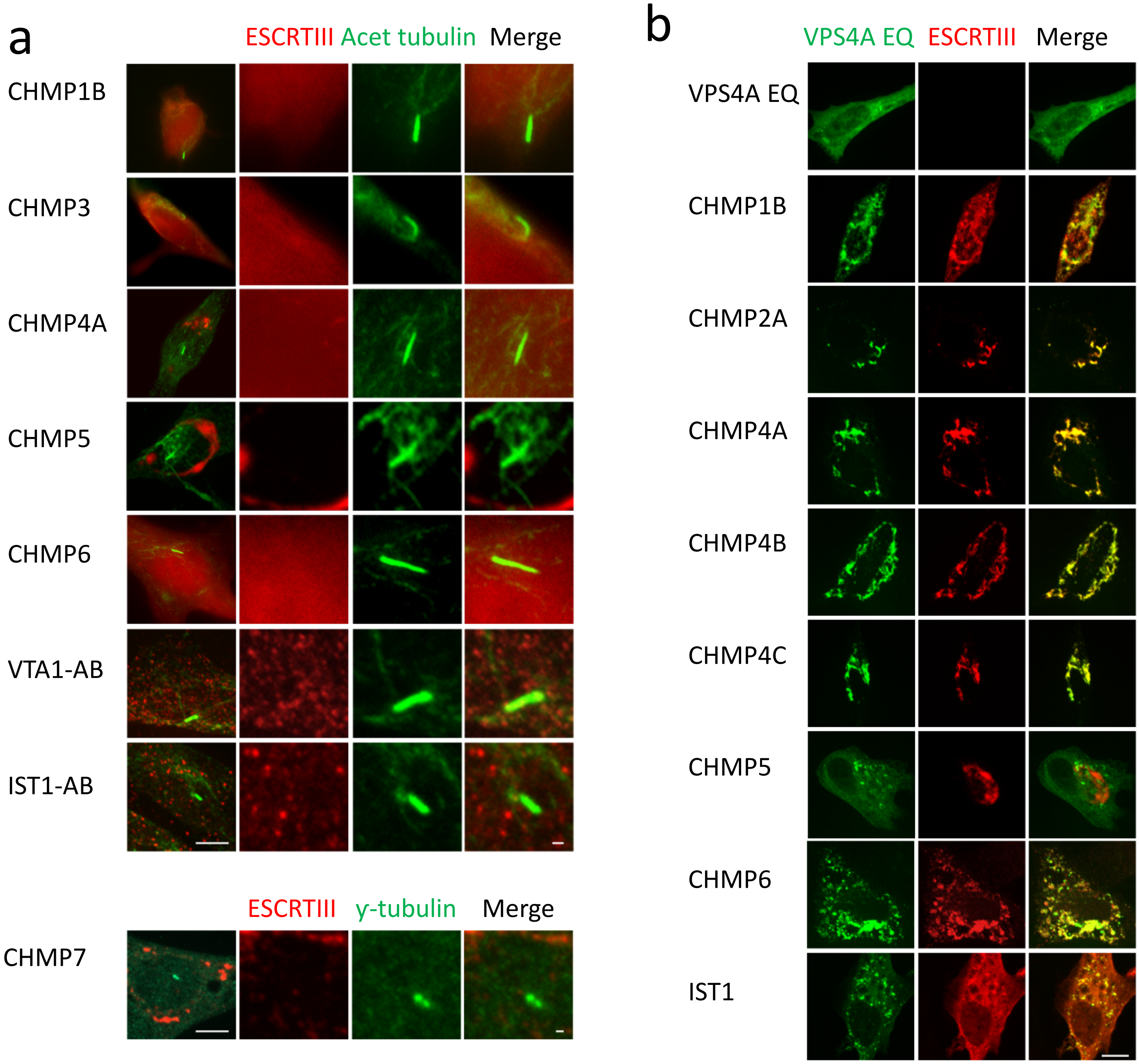

supplementary Fig. 2

ESCRT-III proteins do not localize to centrosome

(a) Top panels: NIH3T3 cells, transfected with fluorescently-tagged ESCRT-III components or immunostained for the endogenous proteins (VTA1, IST1) were stained with anti-acetylated tubulin antibodies. Bottom panel: cells were transfected with myc-CHMP7, fixed and immunostained for myc and γ-tubulin. All cells were imaged using a confocal spinning disk microscope. Maximum projections of representative cells are shown. Left to right: an overlay image (scale, 10 µm), zoomed-in images of ESCRT-III components (red), acetylated tubulin or γ-tubulin (green) alone and an overlay image (scale, 1 µm top panel, 0.2 µm bottom panel). None of the ESCRT-III proteins that were tested were found to be co-localized to the centrosome or the base of the cilium. Images correspond to the graph shown in Fig. 2b. **(b)** Fixed NIH3T3 cells, co-transfected with GFP-VPS4^{EQ} and fluorescently-tagged ESCRT-III components, were immunostained with γ-tubulin antibodies. Cells were imaged using a confocal spinning disk microscope and maximum projections of representative cells are shown. Left to right: VPS4^{EQ} (green), an ESCRT-III component (red), and an overlay. Note that over expression of ESCRT-III perturbed VPS4^{EQ} localization to the centrosome. CHMP1B-HA n=14, mCheery-CHMP2A n=6, mCheery-CHMP4A n=7, mCheery-CHMP4B n=8, mCheery-CHMP4C n=8, GFP-CHMP5 n=6, mCherry-CHMP6 n=8, Flag-CHMP6 n=14, myc-IST1 n=11. Scale, 10 µm.

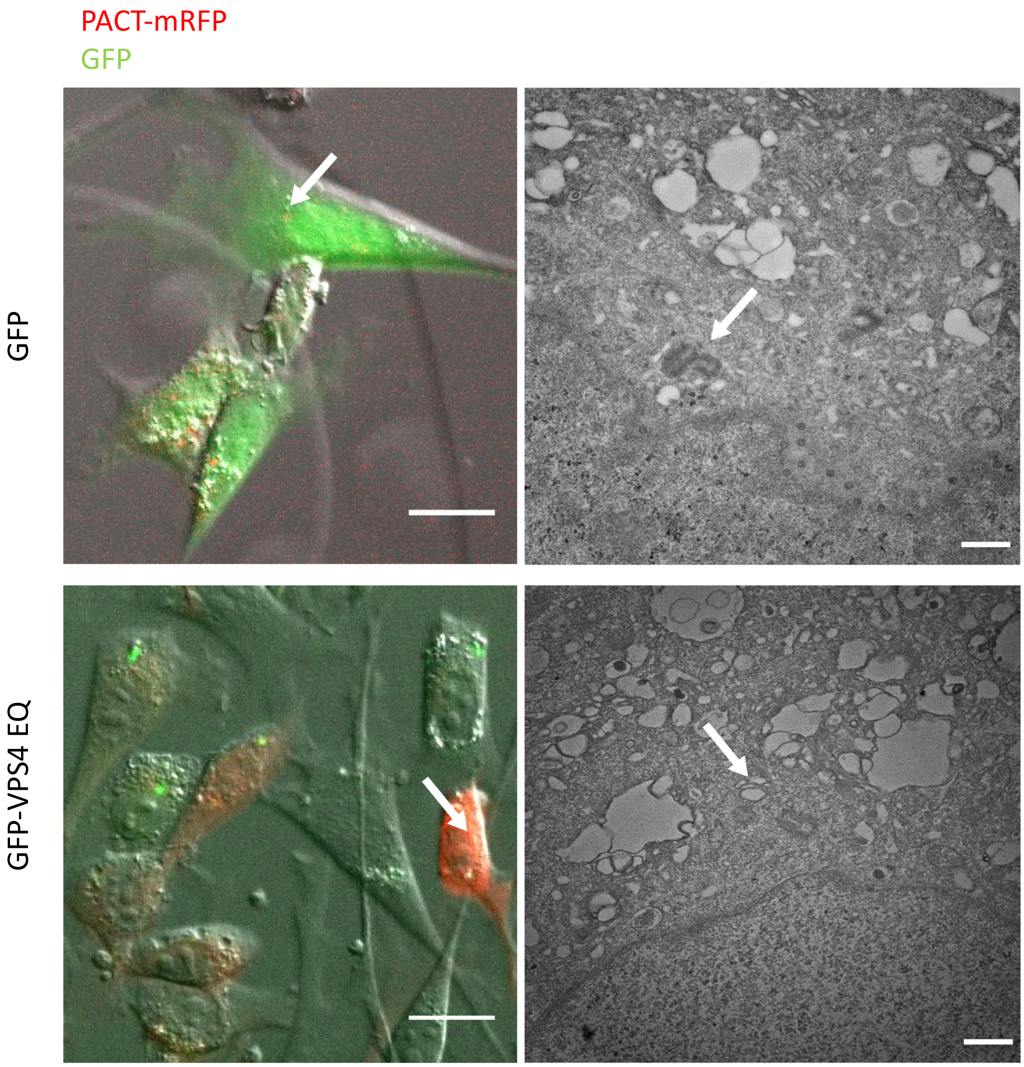

Imaging centrosomes using correlative fluorescence-EM

NIH3T3 cells were plated on gridded coverslips, transfected with PACT-mRFP, and either GFP or GFP-VPS4^{EQ}, fixed and imaged using wide-field microscopy (left panel). Cells expressing both proteins were mapped on the grid. Cells were then subjected to EM sample preparation and serial sectioning as described in material and methods. Serial sections of mapped locations were imaged by EM. Sections that included the centrosome were selected for analysis (right panel). Arrows depict centrosomes in fluorescence and zoomed out EM images that correspond to EM images shown in Fig. 3a. Scale: left panel, 20 µm; right panel, 600 nm.

supplementary Fig. 3

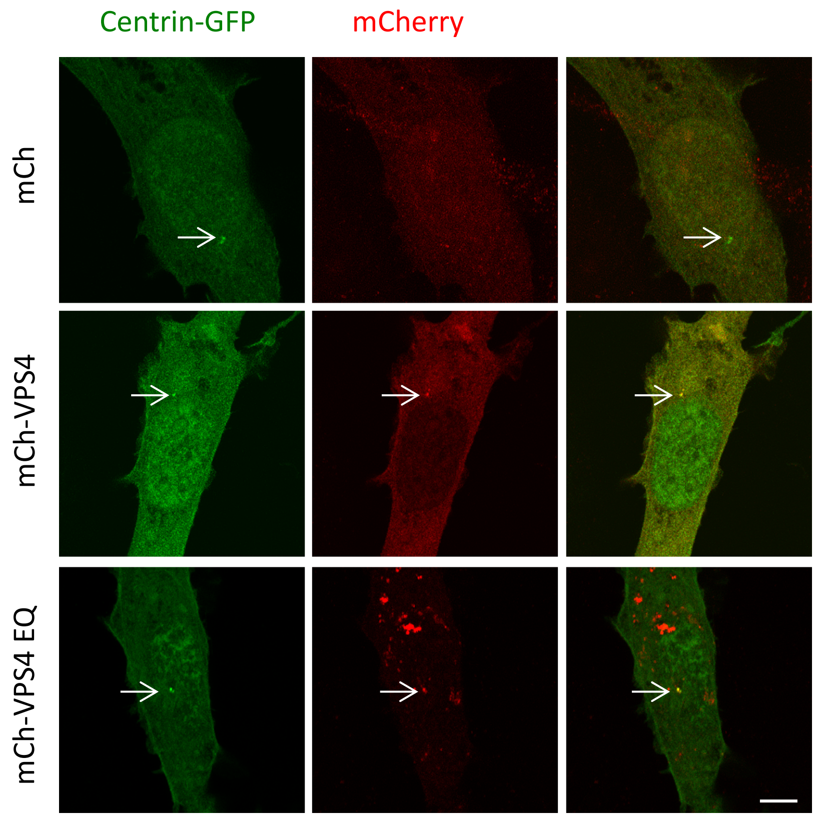

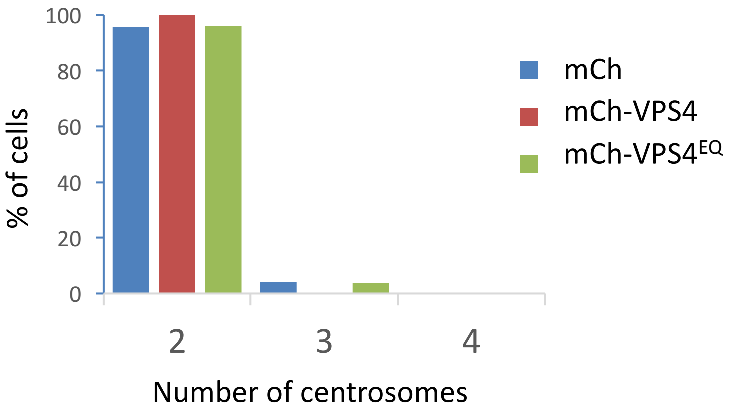

VPS4 expression does not affect centrosome number

NIH3T3 cells were co-transfected with Centrin-GFP (to mark the centrosome) together with mCherry, mCherry-VPS4 or mCherry-VPS4^{EQ}. Cells were fixed and imaged in Airyscan microscope. Maximum intensity projection of representative images from each condition are shown. Number of centrosome in each cell was determined based on Centrin florescence, a summary of the results is shown in the graph below. mCherry n=24 cells, mCherry-VPS4 n=15 cells, mCherry-VPS4^{EQ} n=25 cells. Arrows indicate centrosome. Scale, 5 µm.

supplementary Fig. 4

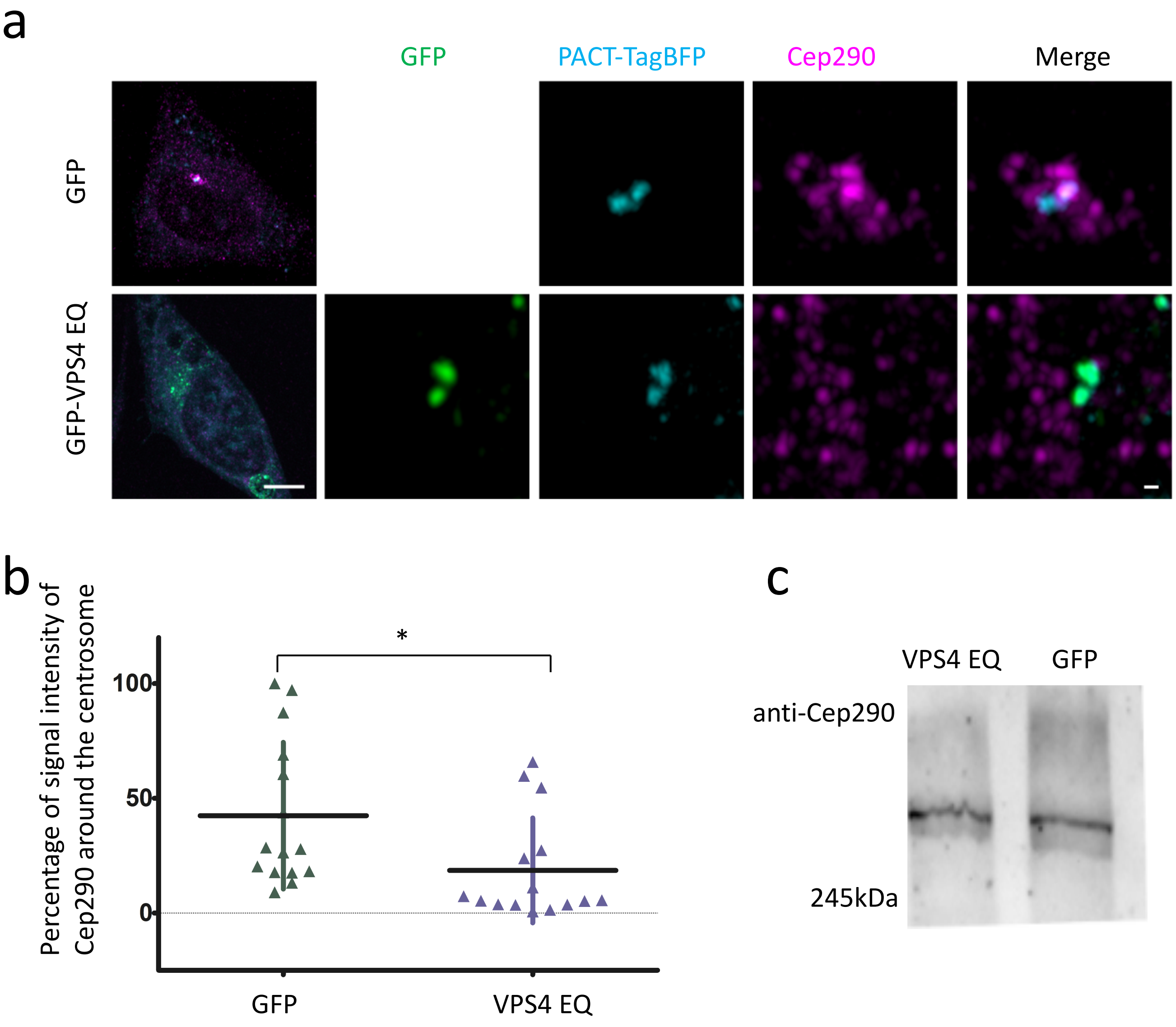

Loss of Cep290 satellites from centrosomes in GFP-VPS4^{EQ} expressing cells

(a) NIH3T3 cells were co-transfected with PACT-TagBFP (blue) together with either GFP or GFP-VPS4^{EQ} (green). Cells were fixed, immunostained with Cep290 antibodies and imaged using Airyscan microscopy. Maximum intensity projections of representative images are shown. Left to right: an overlay image of the entire cell (scale, 5 μm), zoomed-in images of GFP (green), PACT-TagBFP (blue), Cep290 (magenta) and an overlay zoomed-in image (scale, 0.2 μm). **(b)** Quantification of Cep290 signal intensities around the centrosome are shown for GFP or VPS4^{EQ} transfected cells. An area of 0.85μm^2 around the centrosome was measured. Statistical analysis was calculated using a two tail t-test (*p- value ≤ 0.05). GFP n=15, GFP-VPS4^{EQ} n=15. **(c)** Western blot analysis of total Cep290 levels in GFP or VPS4^{EQ} transfected cells.

.

supplementary Fig. 5

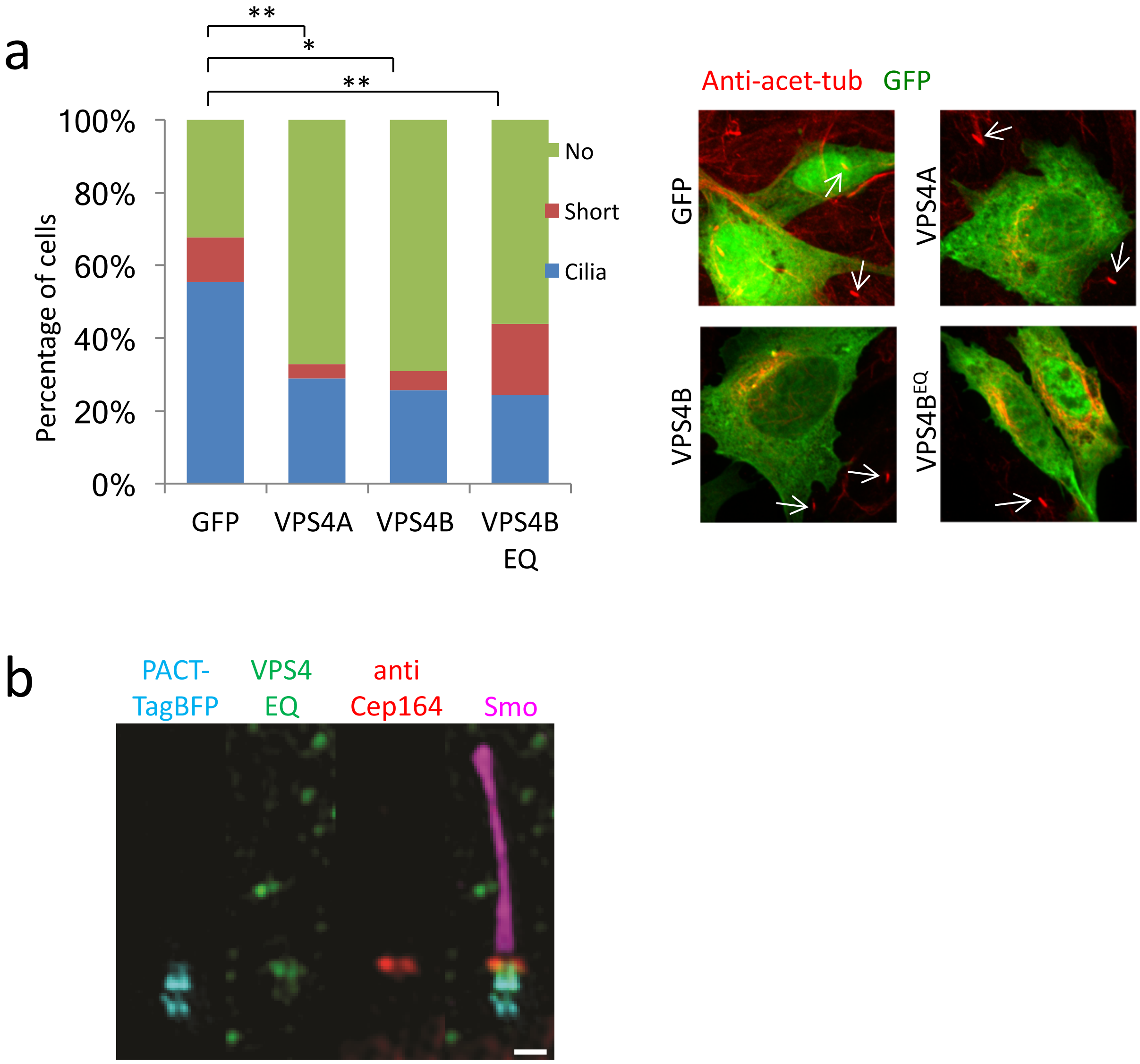

Depletion or overexpression of VPS4 affects cilia formation

(a) Left panel: NIH3T3 cells transfected with the indicated plasmids were fixed, immunostained with anti-acetylated tubulin antibodies and imaged using a spinning disk confocal microscopy. Percentage of ciliated cells in each condition is shown in graph (short cilia ≤ 2 µm, cilia≥ 2 µm). Statistical analysis for normal cilia (≥ 2 µm) was calculated using a one way ANOVA *p- value≤ 0.01, **p- value≤ 0.001). Right panel: maximum intensity projections of representative cells under different conditions are shown. Arrows indicates cilia. GFP n≥700, GFP-VPS4A n=222, GFP-VPS4B n=135, GFP-VPS4B^{EQ} n=158. Scale, 10 µm.**(b)** NIH3T3 cells were transfected with PACT-TagBFP (blue), GFP-VPS4^{EQ} (green) and a cilia-localized membrane protein, TdTomato Smo (magenta). Cells were fixed, immunostained for Cep164 (red) and imaged using SIM. In most cells GFP-VPS4^{EQ} localized as described in Fig. 7f. In a few cells, GFP-VPS4^{EQ} was localized to or near the transition zone, as shown here. Scale, 0.5 µm.

supplementary Fig. 6

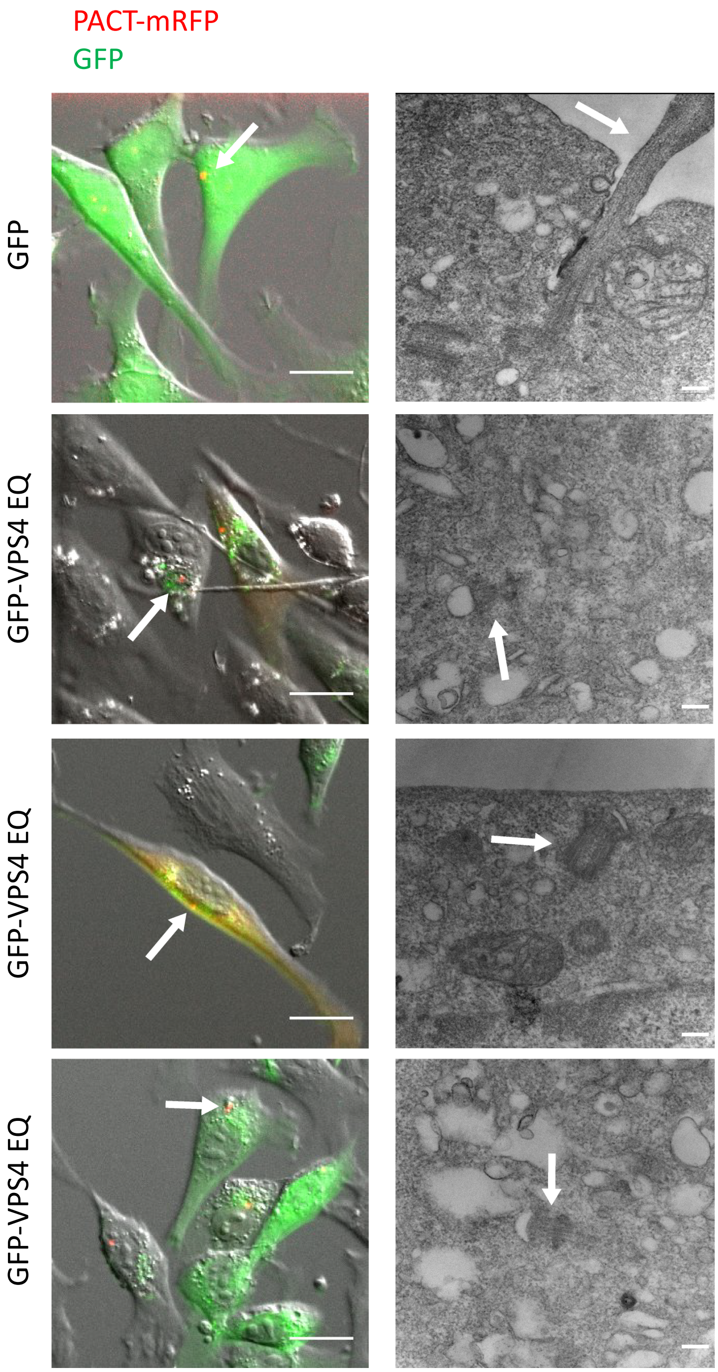

Imaging centrosomes using correlative fluorescence-EM

NIH3T3 cells were plated on gridded coverslips, transfected with PACT-mRFP, and either GFP or GFP-VPS4^{EQ}, fixed and imaged using wide-field microscopy (left panel). Cells expressing both proteins were mapped on the grid. Cells were then subjected to EM sample preparation and serial sectioning as described in material and methods. Serial sections of mapped locations were imaged by EM. Sections that included a centrosome or a cilium were selected for analysis (right panel). Arrows depict centrosomes/cilia in fluorescence and zoomed out EM images that correspond to EM images shown in Fig. 7d. Scale: left panel, 20 µm; right panel, 200 nm.

.

supplementary Fig. 7

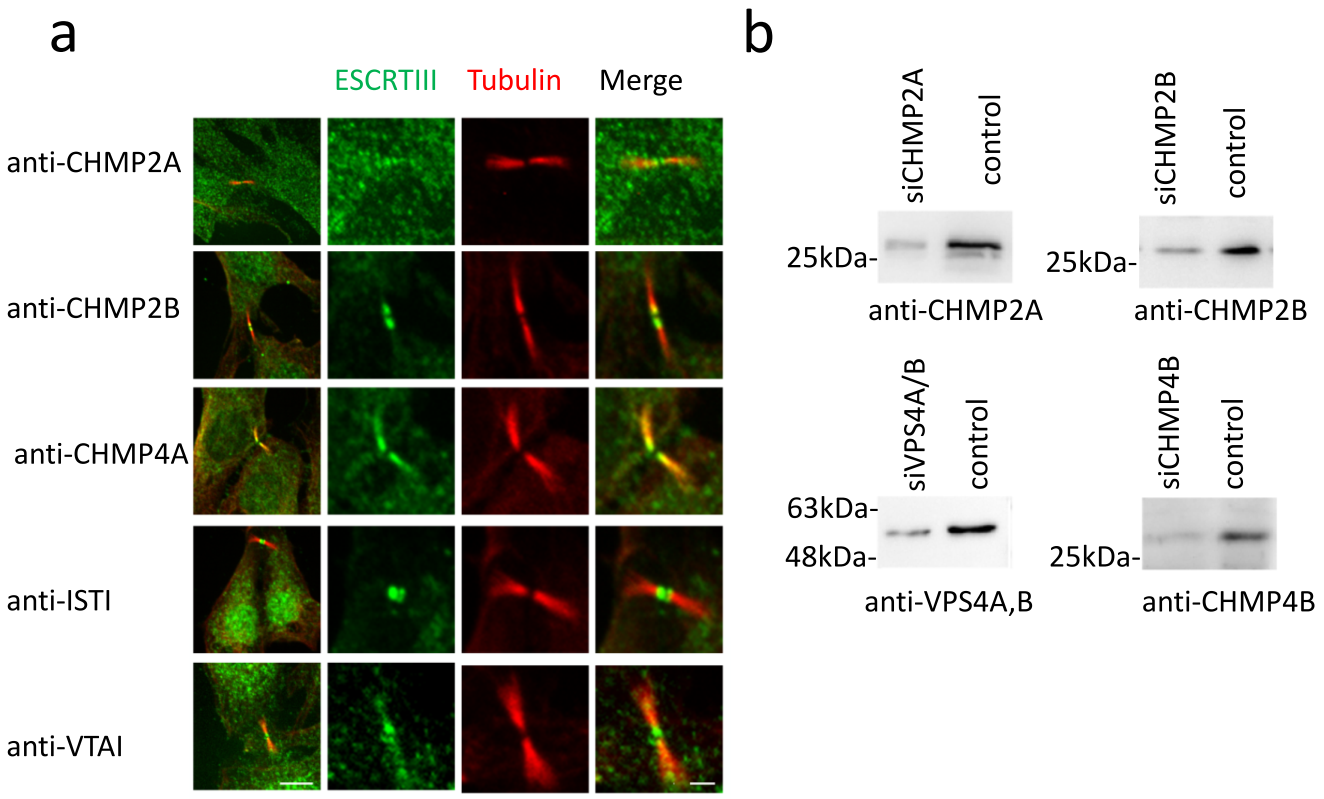

Validation of antibodies and siRNAs

(a) Fixed NIH3T3 cells were immunostained with anti-α-tubulin and the specified ESCRT-III antibodies. Cells were imaged using a confocal spinning disk microscope. Maximum intensity projections of representative cells are shown. Left to right: an overlay image, a zoomed-in image of ESCRT-III (green) or tubulin (red) and a zoomed-in overlay image. Staining of ESCRT-III in the intracellular bridge of cells in late cytokinesis was observed for all antibodies, validating their specificity. CHMP2A n=5, CHMP2B n=6, CHMP4A n=8, VTAI n=7 and ISTI n=6. Scale, 10 µm, zoomed-in image; scale, 2 µm. **(b)** siRNA depletion of ESCRT-III and VPS4 proteins in NIH3T3. Cells were transfected twice, in 24 hour intervals, with the indicated siRNA constructs, lysed and subjected to western blot analysis using the specified antibodies. Equal amounts of total protein were loaded in each lane.

supplementary Fig. 8

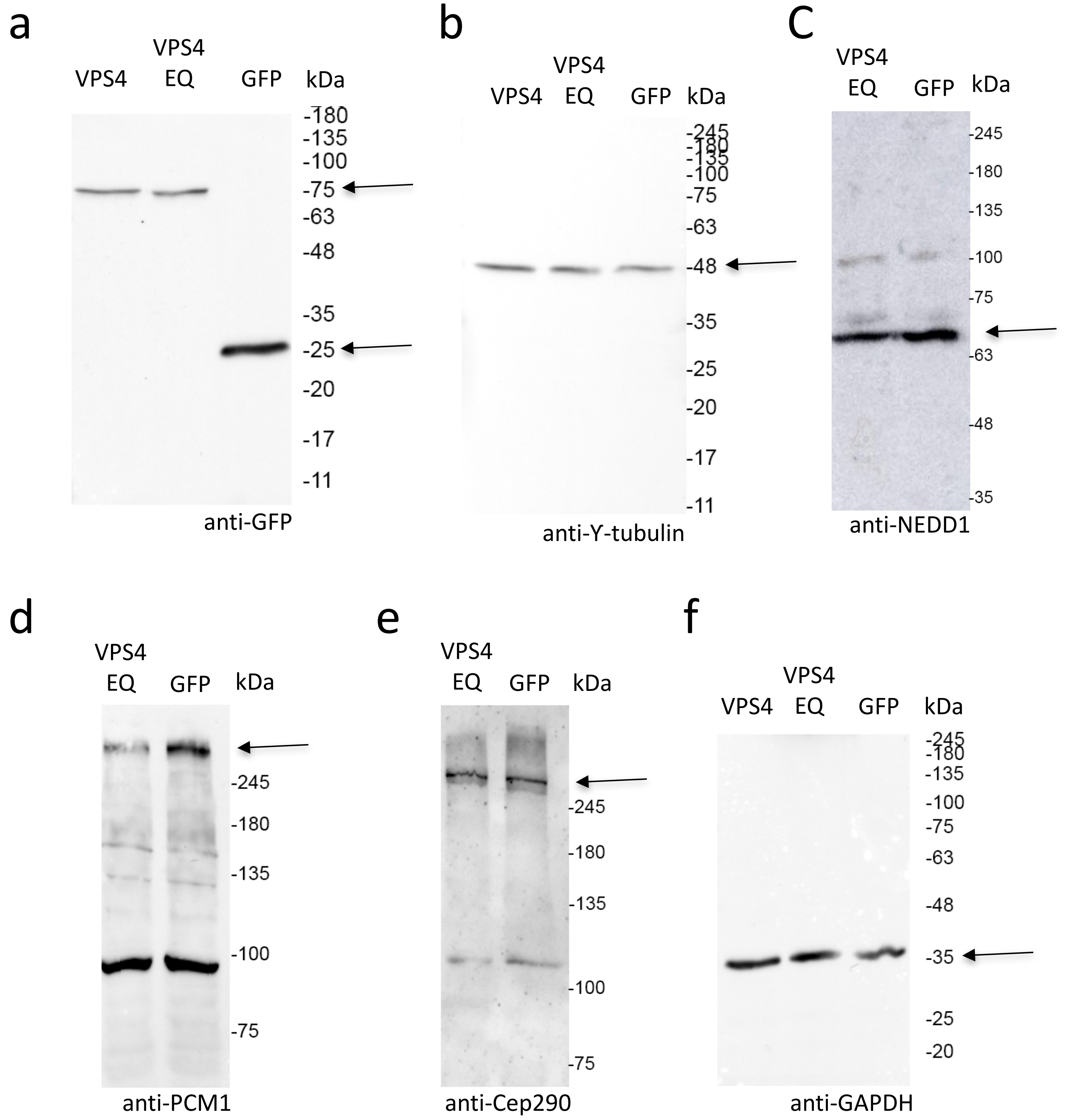

Full length Western blots

NIH3T3 cells transfected with GFP, GFP-VPS4 or GFP-VPS4^{EQ} (as indicated) were harvested and lysed 24 h post transfection and subjected to western blot analysis using the specified antibodies. **(a)** Corresponds to Figure 1b. **(b)** Corresponds to Figure 4d. **(c)** Corresponds to Figure 4e. **(d)** Corresponds to Figure 6c. **(e)** Corresponds to Figure s5c. Equal total protein amounts were loaded. Loading control is shown in **(f)**. Arrows indicate bands shown in main figures.

supplementary Fig. 9
